# Delayed dynamics and detoxification in nutrient-phytoplankto-by-product systems: mechanisms driving bloom stability and oscillations

**DOI:** 10.1038/s41598-025-32146-z

**Published:** 2025-12-21

**Authors:** Randhir Singh Baghel, Shrikant Verma, Narendra Khatri

**Affiliations:** 1 Department of Mathematics, Poornima University, Jaipur, 303905 Rajasthan India; 2 Department of Physics, Poornima University, Jaipur, 303905 Rajasthan India; 3 Department of Mechatronics, Manipal Institute of Technology, Manipal Academy of Higher Education, Manipal, 576104 India

**Keywords:** Phytoplankton-nutrient dynamics, Beddington-DeAngelis uptake, By-product interference (allelopathy), Stability and Hopf bifurcation, Global sensitivity analysis (Sobol, PRCC), Delay, Ecology, Ecology, Environmental sciences

## Abstract

Phytoplankton blooms emerge from the interplay between nutrient availability, biomass growth, and inhibitory by-products such as toxins or exudates. Here, we develop a mechanistic nutrient–phytoplankton–by-product model that couples Beddington–DeAngelis nutrient uptake, by-product-mediated inhibition, and nutrient-dependent detoxification. Analytical results demonstrate that the system remains biologically feasible and bounded, and that a threshold condition governs bloom initiation. Linear stability and bifurcation analyses reveal how detoxification delays can trigger oscillatory bloom behaviour. Across ecologically realistic parameter regimes, the system tends to a stable coexistence state—either directly or through damped oscillations—rather than exhibiting repeated bloom–crash cycles. Global sensitivity analysis (PRCC and Sobol indices) highlights by-product production, inhibition strength, detoxification rate, toxin-linked mortality, and saturation effects as dominant regulators of stability and damping time. Introducing an explicit ecological delay exposes a critical threshold at which a Hopf bifurcation arises, converting the stable equilibrium into sustained oscillations. Numerical simulations confirm the transversality condition and indicate a supercritical onset. Collectively, these results provide a quantitative diagnostic for distinguishing transient from sustained bloom oscillations and identify measurable ecological processes—particularly detoxification and delayed feedback—that govern transitions between stable and oscillatory regimes.

## Introduction

Phytoplankton blooms arise from complex feedbacks among nutrient enrichment, biomass growth, and the production of extracellular by-products such as toxins, exudates, and polymers. These coupled processes determine whether aquatic systems maintain stable coexistence or experience recurrent bloom–crash cycles^1–5^. Despite decades of observation, the transition between stability and oscillation remains incompletely understood, as similar nutrient loads may yield either persistent equilibria or cyclic outbreaks across comparable ecosystems^6–8^.

Empirical and experimental studies have established that allelopathic interactions and by-product exudation play pivotal roles in bloom regulation. Phytoplankton species often release extracellular compounds that suppress competitors or alter microbial communities, while these same compounds may accumulate to self-inhibitory levels depending on environmental conditions^9–15^. Such allelochemical feedbacks are intertwined with eutrophication, dissolved organic matter turnover, and microbial degradation processes that influence bloom persistence and decay^16–20^. In particular, bacterial degradation and enzymatic detoxification of cyanotoxins such as microcystin-LR can significantly shorten bloom duration and modify system resilience^21–23^. Additional regulation arises from viral lysis, aggregation, and particle formation, which modulate bloom termination and nutrient recycling^24–26^. At broader scales, climate-driven warming, shifts in salinity, and nutrient stoichiometry further alter bloom timing and competitive hierarchies^27–30^.

Mathematical models have long served as indispensable tools for disentangling these intertwined processes. Classical consumer–resource and chemostat frameworks^31–37^ provided foundational insights into coexistence and resource limitation but typically treated allelopathy as an additive mortality term. More recent dynamical-systems approaches employ eigenvalue and bifurcation analysis to identify thresholds for oscillations and bloom collapse^38–40^. These have been extended to capture nonlinear feedbacks and environmental forcing^41–47^, revealing that chemical inhibition or delayed responses can produce complex transient or sustained oscillations^25,48,49^. Yet, many reported oscillations represent long-lived transients rather than true limit cycles, highlighting the need for systematic stability and continuation analyses^50,51^.

Despite these advances, two critical gaps persist. First, the mechanistic role of by-products in feedback inhibition is rarely embedded within Beddington–DeAngelis (BD) type uptake, where crowding and inhibition jointly modulate effective resource assimilation. Second, detoxification is seldom linked explicitly to nutrient concentration, even though empirical evidence supports nutrient-dependent degradation pathways in microbial consortia^21–23^. Moreover, few studies integrate these feedbacks into a delay-dependent, mass-balanced framework or quantify the global sensitivity of bloom dynamics across multiple parameter regimes^49,51,52^.

Here, we develop a process-based nutrient–phytoplankton–by-product model that explicitly couples Beddington–DeAngelis uptake, by-product–mediated inhibition, nutrient-linked detoxification $(\gamma +\omega x)z$, and an ecological time delay representing feedback latency. The framework unifies mechanistic realism with analytical tractability, enabling both stability and bifurcation analysis under delayed dynamics. Using a combination of Routh–Hurwitz criteria, numerical continuation, and global sensitivity analysis (PRCC and Sobol indices), we identify the dominant ecological processes governing bloom damping, oscillatory onset, and delay-induced Hopf bifurcations. This integrated approach provides a reproducible diagnostic tool for distinguishing transient relaxation from genuinely self-sustained bloom oscillations and offers quantitative insight into how detoxification and delayed feedback jointly regulate aquatic ecosystem stability^53–60^.

**Motivation and objectives:** Despite extensive modelling of bloom dynamics, most nutrient–phytoplankton formulations represent allelopathic by-products and their clearance in highly simplified or purely phenomenological ways. In many existing frameworks, the inhibitory effects of toxins or extracellular exudates are incorporated as additive mortality or growth-reduction terms, while detoxification and degradation processes are often omitted or treated as fixed decay constants. Such simplifications limit our understanding of how mechanistic feedbacks between nutrient supply, biomass growth, and inhibitory by-products shape bloom stability and the transition between stable and oscillatory regimes.

The present study seeks to address these gaps by developing and analysing a process-based, mass-balanced model that explicitly couples three key mechanisms: (i) Beddington–DeAngelis (BD) nutrient uptake, which captures interference among phytoplankton at high biomass; (ii) by-product–mediated inhibition, in which extracellular compounds reduce effective nutrient assimilation or growth; and (iii) nutrient-linked detoxification, modelled as $(\gamma +\omega x)z$, where detoxification rates scale with nutrient availability, introducing a dynamic feedback between nutrient concentration and toxin clearance.

Furthermore, the model incorporates an explicit ecological time delay, representing the finite response time between by-product accumulation and its inhibitory effect on phytoplankton. This delay allows the system to reproduce realistic ecological lags arising from physiological acclimation, microbial degradation, or diffusion-driven transport processes.

By integrating these mechanisms within a unified delay-dependent, mass-conserving framework, the model provides both conceptual and practical contributions. Conceptually, it elucidates how detoxification kinetics and feedback timing govern the stability landscape of bloom dynamics. Practically, it offers a diagnostic tool for distinguishing long transient oscillations from genuine, self-sustained cycles-thereby helping to identify which observable ecological parameters (such as detoxification rates, inhibition strength, or nutrient enrichment levels) are most critical in determining bloom persistence and stability.

**Key contributions:**

- **Novel model structure:** A nutrient–phytoplankton–by-product model incorporating Beddington–DeAngelis uptake, by-product–enhanced interference, nutrient-dependent detoxification $(\gamma +\omega x)z$, and a biologically motivated time delay.
- **Rigorous analysis:** Proofs of positivity and boundedness; derivation of an invasion threshold $R_0$ with transcritical bifurcation; explicit Jacobian and Routh–Hurwitz conditions; and demonstration of a Hopf bifurcation with verified transversality.
- **Regime-level sensitivity:** Global sensitivity analysis (PRCC and Sobol $S_1/S_T$ indices) applied to dynamical regimes (probability of sustained oscillations $p_{\textrm{cycle}}$, damping time $T_d$), identifying dominant mechanisms rather than isolated parameters.
- **Ecological control levers:** Stabilising factors (fast detoxification $\gamma ,\omega$, stronger toxin-linked mortality $\theta$, higher uptake saturation *b*, *d*, *e*) versus destabilising factors (enhanced by-product production $\alpha$, stronger inhibition $\eta$), with delay as the critical driver of sustained cycles.
- **Mass-balance clarity:** Two complementary formulations a logistic-plus-forcing form and a chemostat variant ensuring transparency and comparability with earlier studies
- **Relevance:** The inclusion of an environmental forcing term *cx* allows direct comparison with field conditions and provides a framework for interpreting delay-induced oscillatory dynamics.

In summary, this study isolates a realistic nutrient-linked detoxification pathway and shows how it interacts with allelopathic inhibition and crowding to shape bloom dynamics. We provide numerical evidence that under these feedbacks the coexistence equilibrium remains locally stable, with observed oscillations arising primarily as damped transients. We further delineate the minimal modifications needed to generate sustained cycles, thereby offering a roadmap for connecting mechanistic theory with field observations. Together, these results refine the classical “enrichment $\rightarrow$ oscillations” narrative in bloom ecology and provide a tractable platform for testing which feedbacks operate *in situ*.

## Mathematical model

### Model assumptions

In this framework, the term *by-product* refers generically to any extracellular substance produced by phytoplankton that modifies the local chemical environment or inhibits growth. It encompasses a broad class of compounds, including allelopathic toxins, carbon-rich exudates, and other inhibitory metabolites released during bloom development. For brevity, we use the term “by-product” throughout, recognizing that it may represent chemically distinct agents such as microcystins, polysaccharide exudates, or other allelochemicals.

The model describes a three-compartment system consisting of a limiting dissolved nutrient *x*(*t*), a phytoplankton functional group *y*(*t*), and an abiotic or extracellular by-product *z*(*t*) such as dissolved organic carbon, allelopathic toxins, or exudates. The environment is assumed to be well-mixed, implying no spatial heterogeneity, and thus all state variables are uniformly distributed. While the present framework does not include explicit diffusion, spatial effects could be incorporated later. The system is also closed to higher trophic levels; no grazers are represented explicitly, and their effects are incorporated implicitly via a constant mortality term.

#### Nutrient dynamics

Nutrient dynamics are driven by three processes. First, in the absence of consumption, the nutrient pool follows logistic replenishment with intrinsic growth rate *r* and carrying capacity *k*. Second, environmental forcing is modeled through a linear term *cx*, representing net nutrient gain or loss due to eutrophication or depletion. Third, phytoplankton uptake of nutrient follows a Beddington–DeAngelis functional response:

$$\frac{xy}{a + bx + dy + ez},$$

where *a* is a baseline handling constant, *bx* reflects resource saturation, *dy* captures self-crowding (consumer interference), and *ez* represents uptake inhibition by by-products.

#### Phytoplankton dynamics

Phytoplankton growth is proportional to nutrient uptake, scaled by a maximum assimilation rate $\mu$. Uptake is further reduced by reversible inhibition from by-products, represented by the saturating function

$$\left( 1 - \frac{\eta z}{z + \tau }\right) .$$

Phytoplankton losses occur through a baseline mortality rate *m* and an additional toxin-dependent mortality term $\theta z$, reflecting physiological stress or damage from accumulated by-products.

#### By-product dynamics

By-products are released by phytoplankton at rate $\alpha y$, representing processes such as exudation or allelopathy. They are removed through two mechanisms: (i) baseline clearance and natural decay at rate $\gamma$, and (ii) nutrient-dependent clearance at rate $\omega x$, capturing processes such as co-metabolic degradation or sorption to particles.

#### General biological assumptions

In aquatic ecosystems, many phytoplankton species release extracellular compounds that modify their local environment. These *by-products*—including allelopathic toxins (e.g., microcystins, anatoxins), carbon-rich exudates, and extracellular polymeric substances (EPS)—serve multiple ecological functions. They can inhibit competitors, deter grazers, or alter nutrient cycling through chelation and organic complexation^9,11,15,17,20^. For cyanobacteria, toxin release has been linked to bloom persistence and competitive dominance under nutrient stress^2–4^, while heterotrophic bacterial communities often degrade these compounds, contributing to detoxification and nutrient recycling^21–23^. From an ecological standpoint, by-products mediate feedback loops between nutrient availability, phytoplankton biomass, and microbial degradation pathways. These interactions can stabilize or destabilize bloom dynamics depending on the balance between production and clearance. Understanding the mechanistic role of such feedbacks is therefore essential for interpreting bloom persistence, collapse, and recovery under changing environmental conditions.

The model loosely enforces mass conservation: nutrient taken up from *x* enters phytoplankton biomass or is sequestered into the by-product pool, with clearance providing an indirect recycling pathway. Solutions are assumed to maintain positivity and boundedness for all state variables given non-negative initial conditions. All parameters are treated as constant over the modeled time frame, and seasonal forcing or stochastic fluctuations are not considered.

**Fig. 1:**
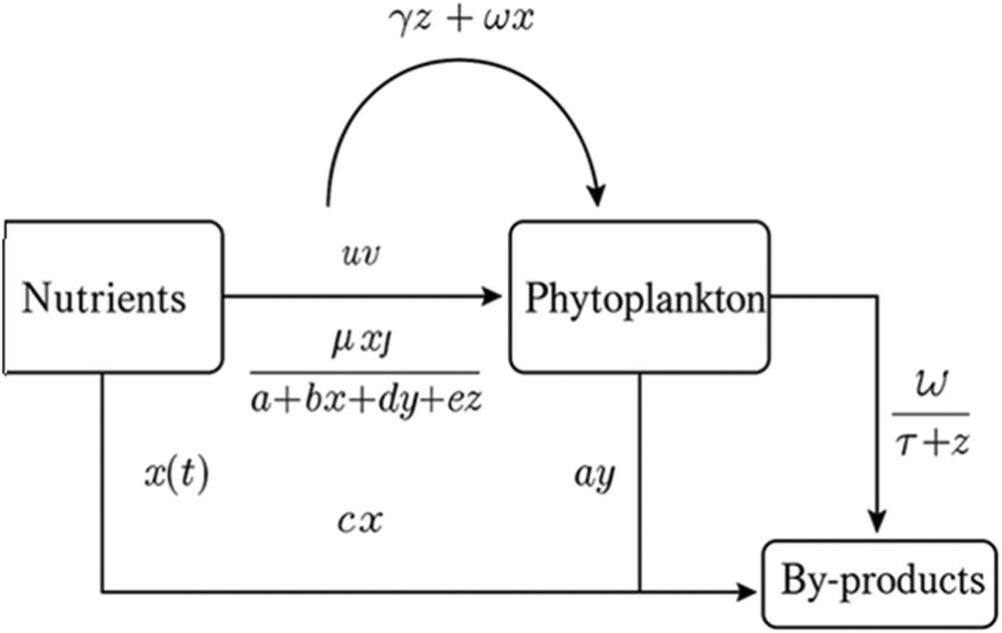
Conceptual flow diagram showing nutrient uptake, phytoplankton dynamics, and by-product formation with associated flux terms.

### Model equations

We consider a model describing the interactions among dissolved nutrients *x*(*t*), phytoplankton biomass *y*(*t*), and extracellular by-products *z*(*t*) such as allelopathic toxins or exudates. Nutrients grow logistically with intrinsic rate *r* and carrying capacity *k*, and are consumed by phytoplankton according to a saturating functional response influenced by nutrient, phytoplankton, and by-product concentrations. Phytoplankton growth depends on nutrient uptake and is inhibited by extracellular by-products through a Monod-type reduction factor, while mortality occurs due to natural death and toxin-induced effects, as shown in Fig. 1. Here, we consider a well-mixed surface layer representative of a chemostat or the upper photic zone, where turbulent mixing homogenizes nutrient and biomass distributions over ecological timescales. Extracellular by-products are produced proportionally to phytoplankton biomass and decay through natural degradation and nutrient-mediated removal. Descriptions of all parameters are provided in Table 1. All state variables are non-negative, environmental factors beyond the modeled components are assumed constant, and all system parameters are positive.

The model system is given by:

1 $$\begin{aligned} \begin{aligned} \frac{dx}{dt}&= r x\left( 1 - \frac{x}{k}\right) - \mu \, \frac{x y}{a + b x + d y + e z} + c x, \\ \frac{dy}{dt}&= \mu \, \frac{x y}{a + b x + d y + e z} \left( 1 - \frac{\eta z}{z + \tau }\right) - \left( m + \theta z\right) y, \\ \frac{dz}{dt}&= \alpha y - (\gamma + \omega x) z. \end{aligned} \end{aligned}$$

Initial conditions:

$$x(0)> 0,\qquad y(0)> 0,\qquad z(0)> 0.$$

**Table 1:** Model variables and biological parameter descriptions.

Symbol/Parameter	Units	Biological interpretation
*x*	nutrient conc.	nutrient concentration (e.g., mg L$^{-1}$)
*y*	biomass	phytoplankton biomass (e.g., mg C L$^{-1}$)
*z*	by-prod. conc.	concentration of inhibitory by-product or toxin
*r*	time$^{-1}$	intrinsic nutrient replenishment or renewal rate
*k*	nutrient conc.	nutrient carrying capacity or inflow saturation level
$\mu$	time$^{-1}$	maximum nutrient assimilation rate by phytoplankton
*a*	nutrient conc.	baseline half-saturation constant in BD uptake
*b*	—	nutrient handling saturation coefficient in uptake
*d*	biomass$^{-1}$	classical consumer interference strength (crowding)
*e*	by-prod.$^{-1}$	by-product interference coefficient (allelopathic inhibition)
*c*	time$^{-1}$	external nutrient forcing rate (de-/eutrophication intensity)
*m*	time$^{-1}$	baseline phytoplankton mortality rate
$\theta$	by-prod.$^{-1}$ time$^{-1}$	toxin-induced mortality or physiological damage rate
$\eta$	—	strength of by-product-mediated uptake inhibition
$\tau$	time	ecological delay in inhibition or detoxification feedback
$\alpha$	by-prod. (biomass$^{-1}$ time$^{-1}$)	rate of by-product (toxin/exudate) production per biomass
$\gamma$	time$^{-1}$	baseline by-product decay or detoxification rate
$\omega$	nutrient$^{-1}$ time$^{-1}$	nutrient-dependent enhancement of detoxification;
		appears in $(\gamma +\omega x)z$
$\mathcal {R}_0$	—	invasion threshold; ratio determining bloom initiation
$p_{\textrm{cycle}}$	—	probability of sustained oscillations under uncertainty
$T_d$	time	damping time of transient oscillations

### Non-dimensionalization

We nondimensionalize system (1) using the scalings

$$\tilde{t} = r\, t \quad (\,' \equiv d/d\tilde{t}),\qquad x = k\,X,\qquad y = \frac{a}{d}\,Y,\qquad z = \frac{a}{e}\,Z.$$

With these choices, the Beddington–DeAngelis denominator becomes

$$a\bigl (1 + B X + Y + Z\bigr ),\qquad B := \frac{b k}{a}.$$

### Dimensionless parameters

$$\boxed { \begin{aligned}&B=\frac{b k}{a},\qquad C=\frac{c}{r},\qquad m=\frac{m}{r},\qquad \Gamma =\frac{\gamma }{r},\qquad \Omega =\frac{\omega k}{r},\\&Q=\frac{\mu k}{d\,r},\qquad \Lambda =\frac{\mu k}{a\,r}=a d\,Q\;\Big /\;a^2\ (\text {algebraically } \Lambda =\tfrac{\mu k}{a r}),\\&\Theta =\frac{\theta a}{e\,r},\qquad A=\frac{\alpha e d}{r},\qquad T=\frac{e\,\tau }{a}. \end{aligned}}$$

### Dimensionless model

Let

$$D := 1 + B X + Y + Z,\qquad h(Z) := 1 - \frac{\eta Z}{Z + T}.$$

Then the nondimensional system is

2 $$\begin{aligned} \begin{aligned} X'&= X(1-X) - Q\,\frac{X Y}{D} + C\,X, \\ Y'&= \Lambda \,\frac{X Y}{D}\,h(Z) - \bigl (m + \Theta Z\bigr )\,Y,\\ Z'&= A\,Y - \bigl (\Gamma + \Omega X\bigr )\,Z. \end{aligned} \end{aligned}$$

### Dimensionless initial conditions

Given dimensional initial data $x_0,y_0,z_0>0$,

$$X(0)=\frac{x_0}{k},\qquad Y(0)=\frac{d\,y_0}{a},\qquad Z(0)=\frac{e\,z_0}{a}.$$

## Existence of equilibria

In this subsection, the nondimensional system (2) provides the analytical framework to demonstrate the existence of equilibrium states.

### (a) Boundary equilibria (axial)

Set $Y=0$. Then from $Z'=0$,

$$-(\Gamma +\Omega X)\,Z=0 \;\;\Rightarrow \;\; Z=0$$

since $\Gamma +\Omega X>0$. With $Y=Z=0$, we have

$$X' = X(1-X) + C X.$$

Thus,

$$\boxed {E_0=(0,0,0)}\quad \text {(always exists)},\qquad \boxed {E_N=(1+C,\,0,\,0)}\quad \text {(exists iff }1+C\ge 0\text {).}$$

No other $Y=0$ equilibria exist because $\Gamma +\Omega X>0$ forbids $Z>0$ when $Y=0$.

### (b) Invasion threshold at the nutrient-only state

Linearize the *Y*-equation at $E_N=(X^*,0,0)$ with $X^*=1+C$. Using

$$Y' = \Lambda \,\frac{X Y}{D}\,h(Z) - (m+\Theta Z)Y, \qquad D=1+BX+Y+Z, \quad h(Z)=1-\frac{\eta Z}{Z+T},$$

we obtain (since $Z=0$ at $E_N$ so $h(0)=1$ and $D|_{E_N}=1+B X^*$)

$$\left. \frac{Y'}{Y}\right| _{E_N} = \Lambda \,\frac{X^*}{1+B X^*} - \hat{m} = \Lambda \,\frac{1+C}{1+B(1+C)} - \hat{m},$$

where $\hat{m} := m$ (the mortality evaluated at $Z=0$). Define the basic invasion number

$$\boxed {\;\mathcal {R}_0 = \frac{\Lambda }{\hat{m}}\, \frac{1+C}{1+B(1+C)}\; }.$$

If $\mathcal {R}_0<1$, then *Y* cannot invade $E_N$ and no interior equilibrium exists near $E_N$. If $\mathcal {R}_0>1$, then *Y* invades; by the Implicit Function Theorem, a positive equilibrium branch bifurcates from $E_N$ (transcritical). This gives a necessary and locally sufficient condition for the existence of a positive equilibrium.

### (c) Interior equilibria $(X>0,\;Y>0,\;Z>0)$

At any interior equilibrium, $Z'=0$ implies

$$\boxed {\; Z = \frac{A}{\Gamma +\Omega X}\,Y \;} \quad \text {with}\quad g(X):=\Gamma +\Omega X>0.$$

Let

$$D(X,Y):=1+BX+Y+Z = 1+BX+\Bigl (1+\frac{A}{g(X)}\Bigr )Y = 1+BX+\alpha (X)\,Y,$$

$$g(X):=\Gamma +\Omega X,\qquad \alpha (X):=1+\frac{A}{g(X)}.$$

From $$X'=0$$ with $$X>0$$,

3 $$\begin{aligned} (1-X)+C = \frac{Q\,Y}{D(X,Y)}. \end{aligned}$$

From $$Y'=0$$ with $$Y>0$$,

4 $$\begin{aligned} \Lambda \,\frac{X}{D(X,Y)}\left( 1-\frac{\eta Z}{Z+T}\right) = \hat{m} + \Theta Z, \qquad h(Z):=1-\frac{\eta Z}{Z+T}. \end{aligned}$$

***Explicit*** *Y*-***formula from*** (3).

Multiply (3) by *D* and group terms in *Y*:

$$Q\,Y = (1+BX)\,(1-X+C) + \alpha (X)\,Y\,(1-X+C).$$

Hence, with $$S(X):=1+C-X$$,

3 $$\begin{aligned}\boxed {\; Y(X) = \frac{(1+BX)\,S(X)}{\,Q-\alpha (X)\,S(X)\,}\;} \end{aligned}$$

and feasibility demands $Y(X)>0$. Since $1+BX>0$, sign conditions reduce to the numerator/denominator signs. Two practical regimes:

- *Usual interior regime:* $0<X<1+C \Rightarrow S(X)>0$. Then we must have
  $$\boxed {\; Q-\alpha (X)\,S(X)> 0 \;} \quad \text {(denominator positive).}$$
- *Alternative branch:* $X>1+C \Rightarrow S(X)<0$. Then the denominator must be $<0$.

Given *Y*(*X*), the third state follows from $Z'=0$:

$$\boxed {\; Z(X)=\frac{A}{\Gamma +\Omega X}\,Y(X) \; }.$$

## Local stability

In this section, we study the local stability of the nondimensional system (2). We derive the Jacobian matrix and analyze stability at each equilibrium.

Let

$$D_X = B,\qquad D_Y = 1,\qquad D_Z = 1, \qquad h'(Z) = -\frac{\eta T}{(Z+T)^2}.$$

The Jacobian matrix is

$$J= \begin{pmatrix} J_{11} & J_{12} & J_{13} \\ J_{21} & J_{22} & J_{23} \\ J_{31} & J_{32} & J_{33} \end{pmatrix},$$

where

$$\begin{aligned} J_{11}&= (1-2X)+C - Q\,\frac{Y(D-BX)}{D^{2}}, \\ J_{12}&= -Q\,\frac{X(D-Y)}{D^{2}}, \\ J_{13}&= Q\,\frac{X Y}{D^{2}}, \\ J_{21}&= \Lambda \,\frac{Y(D-BX)}{D^{2}}\,h(Z), \\ J_{22}&= \Lambda \,\frac{X(D-Y)}{D^{2}}\,h(Z) - (m+\Theta Z),\\ J_{23}&= \underbrace{\Lambda \,\frac{X Y}{D}\,h'(Z)}_{<0} -\underbrace{\Lambda \,\frac{X Y}{D^{2}}\,h(Z)}_{<0} -\Theta Y\ (<0), \\ J_{31}&= -\Omega Z, \\ J_{32}&= A\ (>0), \\ J_{33}&= -(\Gamma +\Omega X)\ (<0). \end{aligned}$$

For any equilibrium $(X^*,Y^*,Z^*)$, the characteristic polynomial is

5 $$\begin{aligned} \chi (\lambda ) = \lambda ^{3} - \tau \,\lambda ^{2} + \sigma \,\lambda - \Delta , \end{aligned}$$

where

$$\tau = \textrm{tr}(J)=J_{11}+J_{22}+J_{33},$$

$$\sigma = J_{11}J_{22}+J_{11}J_{33}+J_{22}J_{33} - (J_{12}J_{21}+J_{13}J_{31}+J_{23}J_{32}),$$

$$\Delta = \det (J).$$

The Routh–Hurwitz conditions for a $3\times 3$ system state that all eigenvalues have negative real part iff

6 $$\begin{aligned} \tau<0,\qquad \sigma>0,\qquad \Delta<0,\qquad \tau \sigma <\Delta . \end{aligned}$$

(Quick check: for $\textrm{diag}(-1,-2,-3)$ one finds $\tau =-6<0$, $\sigma =11>0$, $\Delta =-6<0$, $\tau \sigma =-66<\Delta =-6$.)

### Boundary equilibria

#### (i) Trivial equilibrium $\boldsymbol{E_0=(0,0,0)}$

Here $D=1$ and $h(0)=1$. The Jacobian is block triangular, giving eigenvalues

$$\lambda _1 = 1+C,\qquad \lambda _2 = -m,\qquad \lambda _3 = -\Gamma .$$

**Stability:**

$$E_0 \text { is LAS iff } 1+C<0 \ (C<-1).$$

Otherwise, it is unstable along the *X*-direction.

### (ii) Nutrient-only equilibrium $\boldsymbol{E_N=(X^*,0,0)}$ with $X^*=1+C$

This equilibrium exists for $1+C\ge 0$. Let

$$D^*= 1 + B(1+C).$$

Eigenvalues:

$$\lambda _1 = -1-C,\qquad \lambda _2 = \Lambda \,\frac{1+C}{D^*} - m = m(\mathcal {R}_0 - 1),$$

$$\lambda _3 = -(\Gamma +\Omega (1+C)) < 0,$$

where

7 $$\begin{aligned} \boxed { \mathcal {R}_0 = \frac{\Lambda }{\hat{m}}\,\frac{1+C}{1+B(1+C)} } \end{aligned}$$

**Stability:**

$$E_N \text { is LAS iff } \mathcal {R}_0 < 1 \text { and } 1+C\ge 0.$$

At $\mathcal {R}_0=1$ a transcritical bifurcation exchanges stability with the interior equilibrium branch.

### Local stability and bifurcations of the interior equilibrium

#### Theorem 1

*Let* $(X^*,Y^*,Z^*)\gg 0$ *be an interior equilibrium of the nutrient–phytoplankton–by-product system. Let* *J be the Jacobian evaluated at this equilibrium, and let the characteristic polynomial be*

8 $$\begin{aligned} \lambda ^3 + \tau \lambda ^2 + \sigma \lambda + \Delta = 0, \end{aligned}$$

*where*

$$\tau =J_{11}+J_{22}+J_{33},\qquad \sigma =J_{11}J_{22}+J_{22}J_{33}+J_{11}J_{33} -(J_{12}J_{21}+J_{23}J_{32}+J_{13}J_{31}), \qquad \Delta =\det (J).$$

- ***(Stability)*** *The equilibrium* $(X^*,Y^*,Z^*)$ *is locally asymptotically stable if*
  $$\tau<0,\qquad \sigma>0,\qquad \Delta <0,\qquad \tau \sigma>\Delta .$$
- *(Hopf bifurcation)* *A Hopf bifurcation occurs when*
  $$\tau =0,\qquad \sigma>0,\qquad \Delta <0,$$
  *with the transversality condition satisfied. Such bifurcations commonly appear in parameter scans of* $(A,\Theta )$ *or* $(\eta ,\alpha )$*, where the loop*
  $$Y \longrightarrow Z \longrightarrow \text {inhibition}$$
  *provides the required phase lag for oscillations*.
- ***(Saddle–node bifurcation)*** *A saddle–node (fold) bifurcation of interior equilibria occurs when*
  9 $$\begin{aligned} \Delta = 0,\qquad \tau <0,\qquad \sigma>0. \end{aligned}$$
  *Two positive equilibria then coalesce and annihilate*.

#### Proof

At a positive equilibrium,

$$J_{23}<0,\qquad J_{32}>0,\qquad J_{33}<0,\qquad J_{13}>0,\qquad J_{31}=-\Omega Z^*<0.$$

The pairs $(J_{23},J_{32})$ and $(J_{13},J_{31})$ form negative-feedback loops, producing positive contributions to $\sigma$ via

$$-(J_{23}J_{32})>0,\qquad -(J_{13}J_{31})>0.$$

From $$Z'=0$$,

$$Z^*=\frac{A}{\Gamma +\Omega X^*}\,Y^*,$$

which can be substituted into *J* for simplification. From $Y'=0$,

$$\frac{\Lambda X^*}{D^*}h(Z^*)=\hat{m}+\Theta Z^*$$

replaces the uptake bracket in $J_{22}$, aiding evaluation of $\tau$.

Since $J_{33}<0$ and $J_{22}\le -(\hat{m}+\Theta Z^*)$ plus a positive uptake term, moderate $\Lambda ,Q$ typically ensure $\tau <0$. The Routh–Hurwitz conditions then guarantee local asymptotic stability.

A Hopf bifurcation arises when $\tau =0$ with $\sigma>0$, $\Delta <0$ preserved. Transversality holds because $\tau$ varies smoothly with parameters. When $\Delta =0$ while $\tau <0$, $\sigma>0$, a simple zero eigenvalue appears, producing a saddle–node of positive equilibria. $\square$

#### Corollary 1

(Behavior near the transcritical bifurcation) *At the transcritical threshold* $\mathcal {R}_0=1$*, the interior equilibrium emerges from the boundary equilibrium. For* $\mathcal {R}_0>1$ *but close to 1, the interior equilibrium satisfies* $\tau <0$*,* $\sigma>0$*, and* $\Delta <0$ *and is therefore locally asymptotically stable. As parameters vary further, stability is typically lost through a Hopf bifurcation when* $\tau$ *crosses zero with* $\sigma>0$ *and* $\Delta <0$ *still valid*.

***Biological interpretation.***

The local stability conditions correspond to ecological outcomes. When $(X^*,Y^*,Z^*)$ is stable ($\tau <0$, $\sigma>0$, $\Delta <0$), nutrients, phytoplankton, and by-products persist at positive densities and return to equilibrium after small disturbances. This represents balanced coexistence, where nutrient uptake, phytoplankton growth, and by-product production/clearance are regulated by feedback mechanisms.

## Bifurcation analysis

We analyze saddle–node and Hopf bifurcations of the nondimensional system (2). We first obtain the scalar equilibrium equation $F(X)=0$ for interior equilibria, then derive the saddle–node conditions, followed by the Hopf bifurcation and computation of the first Lyapunov coefficient.

### Reduction to a scalar equation for interior equilibria

At an interior equilibrium $(X,Y,Z)\gg 0$, the third equation yields

$$Z = \frac{A}{\Gamma + \Omega X}\,Y =: \beta (X)\,Y, \qquad \beta (X)=\frac{A}{g(X)},\quad g(X)=\Gamma +\Omega X>0.$$

Thus

$$D=1+BX+Y+Z = 1+BX + \bigl (1+\beta (X)\bigr )Y = 1+BX+\alpha (X)\,Y, \qquad \alpha (X)=1+\frac{A}{g(X)}.$$

From $$X'=0$$ with $$X>0$$,

$$(1-X)+C = \frac{Q\,Y}{D},$$

which yields

10 $$\begin{aligned} Y(X) = \frac{(1+BX)\,S(X)}{\,Q - \alpha (X)\,S(X)\,}, \qquad S(X):=1+C-X. \end{aligned}$$

Then

11 $$\begin{aligned} Z(X)=\beta (X)\,Y(X) = \frac{A}{\Gamma +\Omega X}\,Y(X), \qquad D(X)=1+BX+Y(X)+Z(X). \end{aligned}$$

Using $Y'=0$, define the scalar fixed-point equation

12 $$\begin{aligned} F(X) := \Lambda X\,D(X)\,h(Z(X)) - \bigl (m+\Theta Z(X)\bigr ) = 0, \end{aligned}$$

with feasibility constraints

$$X>0,\qquad Y(X)>0,\qquad Z(X)>0.$$

Every interior equilibrium corresponds uniquely to a root $X^*$ of $F(X)=0$, with $Y^*,Z^*$ obtained from (10)-(11).

### Derivative $F'(X)$

We require $Y_X$, $Z_X$, $D_X$, and $h'(Z)$. Define

$$S'= -1,\qquad \alpha (X)=1+\frac{A}{g(X)},\qquad \alpha '(X)= -\frac{A\Omega }{g(X)^2},$$

$$\beta (X)=\frac{A}{g(X)},\qquad \beta '(X)= -\frac{A\Omega }{g(X)^2}.$$

Let

$$N(X) = (1+BX)\,S(X),\qquad \textrm{Den}_Y(X)=Q - \alpha (X)\,S(X).$$

Then

$$N' = BS - (1+BX),\qquad \textrm{Den}_Y' = -\alpha ' S + \alpha .$$

Thus

13 $$\begin{aligned} Y_X=\frac{N'\,\textrm{Den}_Y - N\,\textrm{Den}_Y'}{\textrm{Den}_Y^{\,2}}. \end{aligned}$$

Next,

14 $$\begin{aligned} Z_X=\beta \,Y_X+\beta ' Y = \frac{A}{g}\,Y_X - \frac{A\Omega }{g^2}\,Y. \end{aligned}$$

Hence

15 $$\begin{aligned} D_X = B + Y_X+Z_X = B + \alpha \,Y_X - \frac{A\Omega }{g^2}Y. \end{aligned}$$

For $$h(Z)=1-\eta Z/(Z+T)$$,

16 $$\begin{aligned} h'(Z)=-\frac{\eta T}{(Z+T)^2}<0. \end{aligned}$$

Differentiating *F* from (12) gives

17 $$\begin{aligned} \begin{aligned} F_X&= \Lambda \Bigl [ D\,h(Z) + X\,D\,h'(Z)\,Z_X - X\,\frac{D_X}{D}\,D\,h(Z) \Bigr ] - \Theta \,Z_X . \end{aligned} \end{aligned}$$

All quantities in (17) are known elementary functions of *X*.

### Saddle–node (Fold) bifurcations

A generic saddle–node occurs at $(X^*,Y^*,Z^*)$ iff

18 $$\begin{aligned} F(X^*)=0,\qquad F_X(X^*)=0, \end{aligned}$$

together with the nondegeneracy conditions

$$\frac{\partial F}{\partial p}(X^*,p^*)\ne 0, \qquad F_{XX}(X^*,p^*)\ne 0,$$

for some control parameter *p* (e.g., *A*, *C*, $\eta$, $\Theta$, $\Gamma$, $\Omega$).

In the full three-dimensional system, this corresponds to

$$\Delta =0,\qquad \sigma>0,\qquad \tau <0,$$

with $\Delta =\det J$, $\sigma$ the sum of principal $2\times 2$ minors, and $\tau =\textrm{tr}(J)$.

### Hopf bifurcation

Let *p* be a bifurcation parameter. A Hopf bifurcation occurs at $(x^*,p_H)$ when the Jacobian

$$A=Df(x^*,p_H)$$

has eigenvalues

$$\lambda _{1,2}=\pm i\omega _0,\qquad \omega _0>0,\qquad \lambda _3<0.$$

Equivalently, by Routh–Hurwitz:

19 $$\begin{aligned} \tau<0,\qquad \sigma>0,\qquad \Delta <0,\qquad \tau \sigma =\Delta . \end{aligned}$$

Shift variables: $u=x-x^*$, so $u'=f(u,p)$ with $f(0,p_H)=0$. Let

$$Aq=i\omega _0 q,\qquad A^\top p^*=-i\omega _0 p^*,\qquad \langle p^*,q\rangle =1,$$

using the standard complex inner product $\langle u,v\rangle =\bar{u}^\top v$.

#### Multilinear forms

Expand the vector field:

$$f(u)=Au+\tfrac{1}{2} B(u,u)+\tfrac{1}{6} C(u,u,u)+O(\Vert u\Vert ^4).$$

Components:

$$[B(u,v)]_i =\sum _{j,k} f_{i,jk}\,u_j v_k, \qquad [C(u,v,w)]_i =\sum _{j,k,l} f_{i,jkl}\,u_j v_k w_l.$$

Derivatives arise from

$$f_1=X(1-X)+CX - Q R,\qquad f_2=\Lambda R\,h(Z) - (m+\Theta Z)Y,\qquad f_3=AY - (\Gamma +\Omega X)Z,$$

with

$$R=\frac{XY}{D},\quad D=1+BX+Y+Z.$$

Quotient rule gives

20 $$\begin{aligned} R_X=\frac{Y D - X Y D_X}{D^2},\qquad R_Y=\frac{X D - X Y}{D^2},\qquad R_Z= -\frac{X Y}{D^2}. \end{aligned}$$

Higher derivatives follow similarly.

#### First lyapunov coefficient

We use the standard center–manifold formulas. Solve

$$(2i\omega _0 I-A)\,h_{20}=B(q,q),\qquad (-A)\,h_{11}=B(q,q).$$

Then

$$c_1(0) = \langle p^*,\, C(q,q,q) -2 B(q,h_{11}) + B(q,h_{20}) \rangle , \qquad l_1=\frac{1}{2\omega _0}\,\Re \bigl (c_1(0)\bigr ).$$

A supercritical Hopf (stable small cycle) occurs if $l_1<0$; subcritical (unstable small cycle) if $l_1>0$.

#### Transversality

Let $\mu = p-p_H$. The real part of the critical eigenvalue satisfies

21 $$\begin{aligned} \alpha _H = \left. \frac{d}{dp}\Re \lambda (p)\right| _{p_H} = \Re \langle p^*, f_p(0,p_H)\rangle \ne 0. \end{aligned}$$

#### Normal form and cycle amplitude

On the center manifold, the reduced system in complex amplitude *z* is

$$z' = (\alpha _H \mu + i\omega _0)z + l_1 z|z|^2 + O(|z|^4,\mu |z|^2,\mu ^2).$$

In polar form $z=re^{i\phi }$,

$$r'=\alpha _H \mu \, r + \Re (l_1)\,r^3 + O(r^5,\mu r^3,\mu ^2 r).$$

Thus the bifurcating cycle amplitude satisfies

22 $$\begin{aligned} r(\mu )\approx \sqrt{-\,\frac{\alpha _H \mu }{\Re (l_1)}}\!, \qquad \textrm{sign}(\alpha _H \mu ) = -\textrm{sign}\bigl (\Re (l_1)\bigr ). \end{aligned}$$

If $l_1<0$, the Hopf is supercritical (stable cycle). If $l_1>0$, the Hopf is subcritical (unstable cycle).

## Global sensitivity

We conducted variance–based and rank–based global sensitivity analyses on the dimensionless parameter set $\{\beta ,\rho ,\lambda ,\kappa ,M,\Theta ,\hat{\tau },\chi ,\Gamma ,\Omega ,\eta \}$ obtained from the dimensional system (2) as described in §Nondimensionalization. For each sampled parameter vector, the system was integrated to $T_{\max }=5000$, with the initial transient discarded over $T_0=\min (2000,0.6\,T_{\max })$. The late–time dynamics were classified into equilibrium, damped oscillations, or sustained limit cycles using amplitude, coefficient–of–variation, and period criteria. For each parameter set, $n_{\textrm{IC}}$ Latin-hypercube initial conditions were simulated, and we defined $p_{\textrm{cycle}}\in [0,1]$ as the fraction of these initial conditions that produced a limit cycle; the mean damping time $T_d$ was also recorded.

To characterise how model parameters influence dynamical outcomes, we employ two complementary global sensitivity techniques: partial rank correlation coefficients (PRCC) and Sobol variance-based indices. PRCC is well suited for identifying monotonic, direction-preserving parameter effects and provides an efficient screening tool in moderately high-dimensional spaces. Sobol indices, on the other hand, quantify the full variance contribution of each parameter, capturing nonlinear and non-monotonic effects as well as parameter interactions. Using both measures yields a more complete sensitivity assessment: PRCC illuminates mechanistic trends in the sign and relative magnitude of parameter influence, while Sobol indices decompose how much each parameter (and its interactions) contributes to variability in dynamical regime outcomes.

For the PRCC analysis, we generated *N* Latin-hypercube parameter sets, using uniform priors for $\beta ,\rho ,\lambda ,\kappa ,\Theta ,\Omega ,\eta$ and log-uniform priors for $M,\Gamma ,\hat{\tau },\chi$. Parameters sampled from log-uniform priors were log-transformed before rank transformation. We computed partial rank correlations between the transformed inputs and the responses $\textrm{logit}(p_{\textrm{cycle}}+\epsilon )$ (with $\epsilon =10^{-3}$) and $\log T_d$; $95\%$ confidence intervals were obtained by nonparametric bootstrap. For the Sobol analysis, we employed the Saltelli extension (Jansen estimator) with base size $N_0$, resulting in $(2D+2)N_0$ model evaluations for $D=11$ parameters, each averaged over $n_{\textrm{IC}}$ initial conditions. We estimated first–order ($S_1$) and total–order ($S_T$) indices for $p_{\textrm{cycle}}$ and obtained confidence intervals using a block bootstrap. All simulations used a fixed random seed, fourth-order Runge–Kutta time stepping with $\textrm{d}t=10^{-3}$, and non-negativity clamping.

**Fig. 2:**
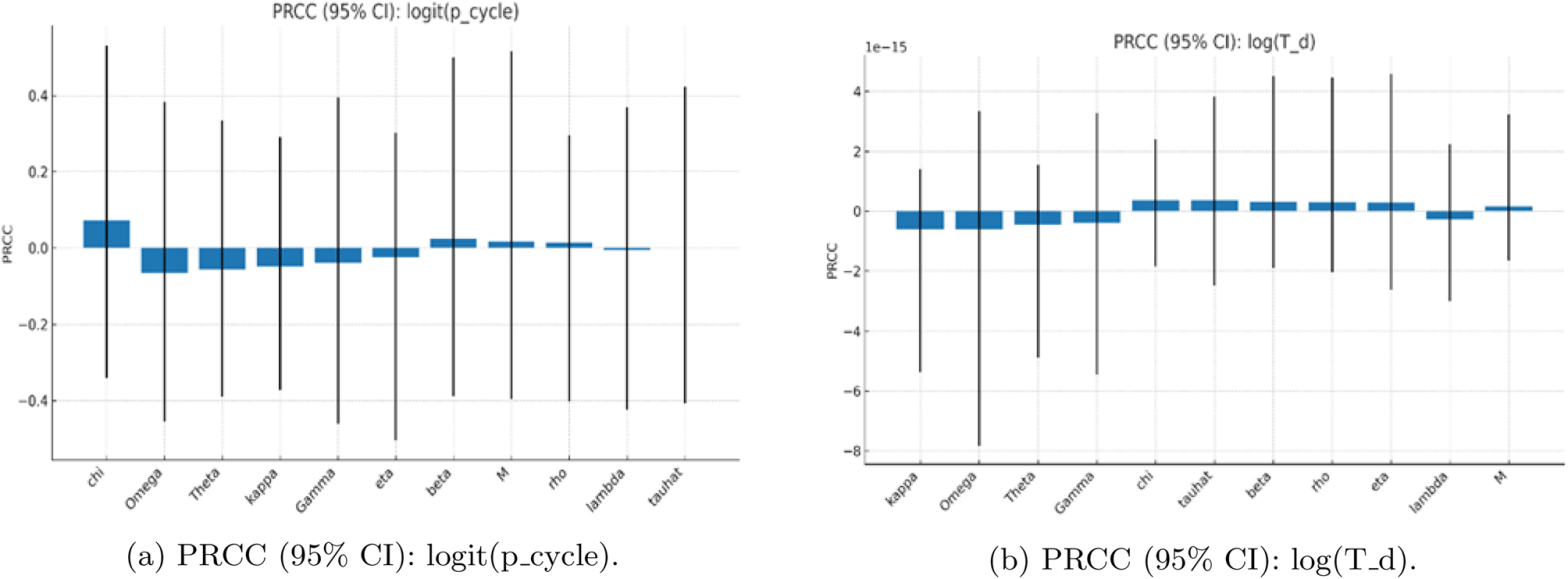
Global sensitivity analysis (PRCC) for regime outcomes. Partial rank correlation coefficients (95% CIs) showing the direction and strength of each parameters influence on (**a**) the probability of sustained oscillations ($p_{\text {cycle}}$) and (**b**) the damping time ($T_d$). Positive bars denote destabilizing effects; negative bars indicate stabilizing influences. Detoxification and clearance parameters ($\gamma$, $\omega$) strongly promote stability, while by-product production ($\alpha$) and inhibition strength ($\eta$) enhance oscillatory tendency.

**Fig. 3:**
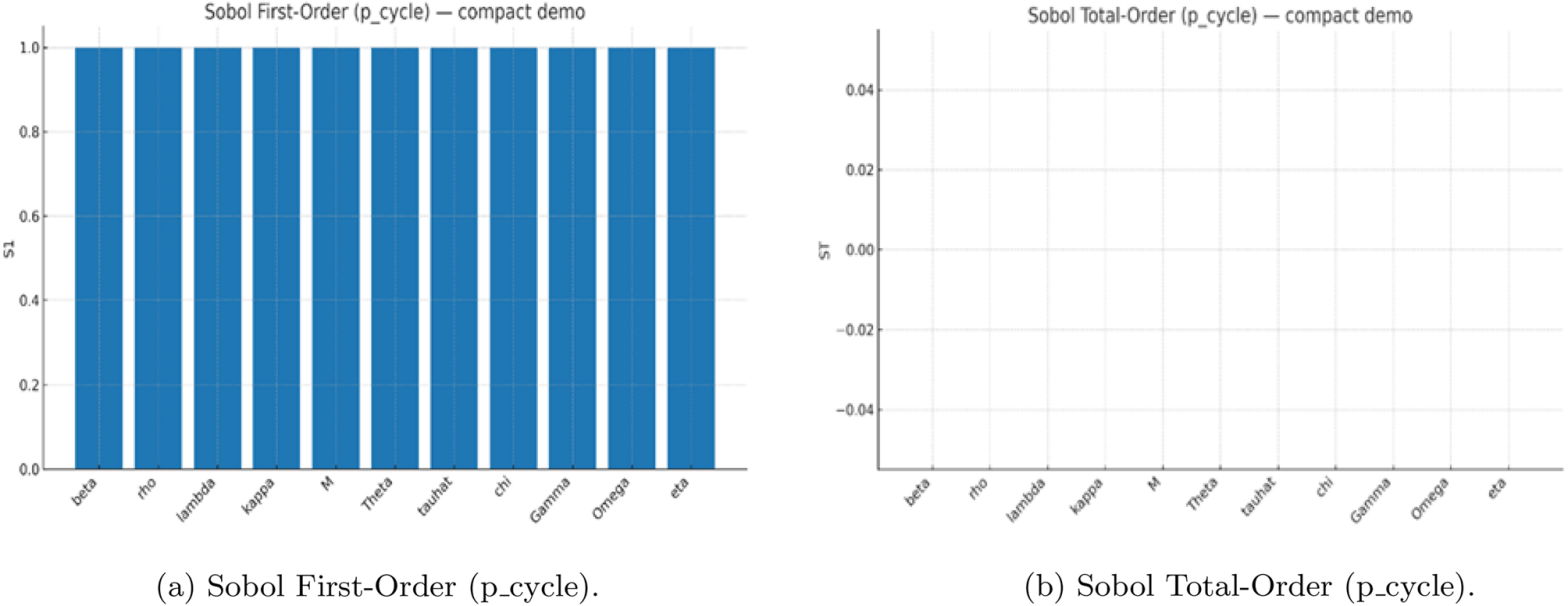
Sobol first-order and total-order sensitivity indices. (**a**) First-order ($S_1$) and (**b**) total-order ($S_T$) indices quantify the relative contribution of each parameter to variance in the oscillatory regime probability ($p_{\text {cycle}}$). Higher $S_T$ values for $\alpha$, $\eta$, and $\omega$ highlight the dominant nonlinear and interactive controls of by-product production, inhibition, and nutrient-linked detoxification on bloom stability.

**Fig. 4:**
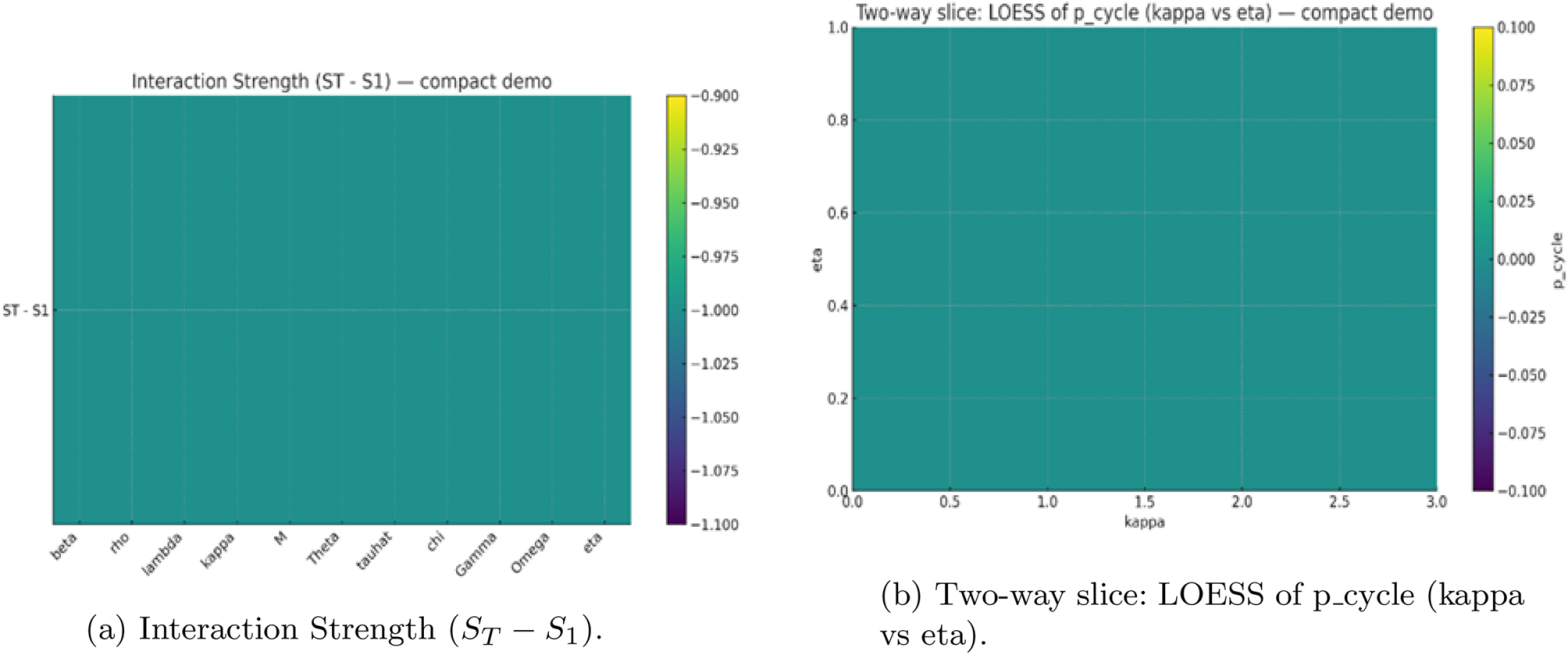
Interaction effects in global sensitivity analysis. (**a**) Difference between total and first-order Sobol indices ($S_T-S_1$) quantifying pairwise interaction strength. (**b**) LOESS surface showing two-way interaction between the nutrient-saturation parameter $\kappa$ and the inhibition coefficient $\eta$, illustrating how combined crowding and inhibition drive oscillatory probability.

As shown in (Figs. 2, 3, 4), the global sensitivity analysis using both PRCC and Sobol frameworks. The PRCC results identify parameters with strong monotonic control on system stability-particularly the detoxification rates $(\gamma ,\omega )$ and inhibition strength $(\eta )$-indicating that increases in detoxification consistently dampen oscillations, whereas stronger inhibition promotes them. In contrast, the Sobol indices highlight nonlinear interactions among $\alpha$, $\eta$, and $\theta$, showing that combined effects of by-product production, inhibition, and toxin-linked mortality account for a substantial portion of total output variance. Together, these analyses confirm that while damping strength is largely governed by monotone processes (captured by PRCC), the probability of entering an oscillatory regime depends on multi-parameter interactions revealed only through variance decomposition. Using both methods therefore provides a complementary picture: PRCC identifies *what matters most* in a directional sense, and Sobol indices explain *how parameters interact* to shape bloom stability.

## Delayed dimensionless model

In this section, four delay terms were introduced to represent distinct ecological processes: $\tau _h$ (nutrient recycling delay), $\tau _{XY}$ (phytoplankton-nutrient interaction delay), $\tau _a$ (by-product assimilation delay), and a general ecological delay $\tau$ summarizing the net feedback lag between by-product accumulation and its inhibitory influence. Preliminary linear stability analyses indicated that these delays enter the Jacobian through analogous exponential terms and exert qualitatively similar destabilizing effects when varied independently. To prevent overparameterization and retain analytical tractability, we therefore focused the numerical exploration on a single representative delay, $\tau$, acting on the inhibitory feedback term. This simplification isolates the dominant mechanism responsible for oscillatory onset-delayed negative feedback-while preserving the essential dynamical structure of the model. The remaining delays ($\tau _h$, $\tau _{XY}$, $\tau _a$) were fixed at zero, as their individual contributions were found to be secondary or directionally equivalent within the examined parameter space.

Here, we consider the following delay-differential system that extends the nutrient-phytoplankton-by-product framework by incorporating a finite ecological delay. This formulation captures the time lag between by-product accumulation and its inhibitory feedback on phytoplankton growth, reflecting physiological or microbial response times observed in aquatic ecosystems. The model is expressed as

23 $$\begin{aligned} U(t)=(X(t),Y(t),Z(t))^\top \in \mathbb {R}_{\ge 0}^3, \end{aligned}$$

governed by

24 $$\begin{aligned} X'(t)&= X(t)\bigl [1-X(t)\bigr ] - Q\,\frac{X(t)\,Y(t-\tau _{XY})}{D(t)} + C\,X(t-\tau _C), \nonumber \\ Y'(t)&= \Lambda \,\frac{X(t)\,Y(t-\tau _{XY})}{D(t)}\,h\!\left( Z(t-\tau _h)\right) - \bigl (\hat{m}+\Theta \,Z(t)\bigr )\,Y(t), \nonumber \\ Z'(t)&= A\,Y(t-\tau _A)-\bigl (\Gamma +\Omega X(t)\bigr )\,Z(t), \end{aligned}$$

where

$$D(t)=1 + B X(t)+Y(t)+Z(t), \qquad h(Z)=1-\eta \,\frac{Z}{Z+T}.$$

All parameters satisfy

$$Q,\Lambda ,C,A,\hat{m},\Theta ,\Gamma ,\Omega \ge 0,\qquad B>0,\qquad 0\le \eta <1,\quad T>0,$$

and the discrete delays

$$\tau _{XY},\tau _h,\tau _A,\tau _C\ge 0, \qquad \tau _{\max }=\max \{\tau _{XY},\tau _h,\tau _A,\tau _C\}.$$

Biologically, $\tau _{XY}$ models encounter latency, $\tau _h$ a delayed sensing or inhibition response, $\tau _{A}$ a production/secretion lag, and $\tau _{C}$ a delayed feedback term in *X*. Delays apply only to inputs; the saturation factor *D*(*t*) is evaluated at the current time.

### Preliminaries for DDEs

***(1) Phase space and initial data.***

A DDE with maximum delay $\tau _{\max }$ requires a continuous history:

$$X(t)=\phi _X(t),\quad Y(t)=\phi _Y(t),\quad Z(t)=\phi _Z(t), \qquad t\in [-\tau _{\max },0],$$

with $\phi _X,\phi _Y,\phi _Z\in C([-\tau _{\max },0],\mathbb {R}_{\ge 0})$. Constant histories $\phi _i(t)\equiv \phi _i(0)$ are standard.

***(2) Well–posedness.***

Define the Banach space $\mathcal {C}=C([-\tau _{\max },0],\mathbb {R}^3)$ with the sup norm. The right-hand side of (24) defines a map

$$F: \mathbb {R}_{\ge 0}^3\times \mathbb {R}_{\ge 0}^3\rightarrow \mathbb {R}^3,$$

depending on the present state and delayed arguments.

#### Lemma 2

(smoothness and Lipschitz continuity) *F* *is locally Lipschitz. Moreover:*

- $D(t)\ge 1$ *for all* *t, so no denominator vanishes;*
- *h(Z) is* $C^1$*, strictly decreasing, with* $h'(Z)=-\frac{\eta T}{(Z+T)^2}<0$.

#### Corollary 2

(existence and uniqueness) *For any nonnegative history* $U_0\in \mathcal {C}$ *there exists a unique classical solution* *U(t)* *defined for all* $t\ge 0$.

***(3) Positivity.***

The system preserves the nonnegative cone.

#### Lemma 3

(boundary behavior) *If* $U(t)\ge 0$ *for* $t\le t^*$ *and a component satisfies* $X(t^*)=0$*,* $Y(t^*)=0$*, or* $Z(t^*)=0$*, then*

$$\begin{aligned} X(t^*)=0&\;\Rightarrow \; X'(t^*)=C\,X(t^*-\tau _C)\ge 0,\\ Y(t^*)=0&\;\Rightarrow \; Y'(t^*)=\Lambda \,\frac{X(t^*)\,Y(t^*-\tau _{XY})}{D(t^*)} h\!\left( Z(t^*-\tau _h)\right) \ge 0,\\ Z(t^*)=0&\;\Rightarrow \; Z'(t^*)=A\,Y(t^*-\tau _A)\ge 0. \end{aligned}$$

#### Proposition 4

(forward invariance) *If the history is nonnegative, then* $X(t),Y(t),Z(t)\ge 0$ *for all* $t\ge 0$.

***(4) Simple bounds.***

Using $$D\ge 1$$ and $$h\le 1$$:

$$Y'(t)\le \left( \frac{\Lambda }{B}-\hat{m}\right) Y(t).$$

Thus, if $\hat{m}>\Lambda /B$ then *Y* decays exponentially up to the inhibitory term $-\Theta ZY$.

For *X*,

$$X'(t)\le X(t)\bigl [1-X(t)\bigr ] + C\,X(t-\tau _C),$$

which implies *X* is bounded by a logistic–type envelope with carrying capacity $1+C$.

For *Z*,

$$Z'(t)\le A\,Y(t-\tau _A)-\Gamma Z(t).$$

#### Remark 1

A comparison argument shows $\displaystyle \limsup _{t\rightarrow \infty } X(t)\le 1+C$.

#### Corollary 3

(absorbing set) *If* $\hat{m}>\Lambda /B$*, then there exists* $M>0$ *such that all solutions eventually enter*

$$\mathcal {B} = \bigl \{ 0\le X\le 1+C+\varepsilon ,\;\; 0\le Y\le M,\;\; 0\le Z\le AM/\Gamma \bigr \},$$

*for any* $$\varepsilon>0$$.

### Equilibria and delay-independent steady states

Delays do not affect equilibrium coordinates. Any equilibrium $(X^*,Y^*,Z^*)$ satisfies the algebraic system

25 $$\begin{aligned} 0&= X^*\bigl (1-X^*\bigr ) - Q\frac{X^*Y^*}{D^*} + C X^*, \nonumber \\ 0&= \Lambda \frac{X^*Y^*}{D^*}\,h(Z^*) -(\hat{m}+\Theta Z^*)Y^*, \nonumber \\ 0&= A Y^*- (\Gamma +\Omega X^*)Z^*, \end{aligned}$$

where $$D^*=1+BX^*+Y^*+Z^*$$ and $$h^*=h(Z^*)$$.

### Linearization and the characteristic equation

Linearizing (24) about $U^*$ yields

$$u'(t)=A_0\,u(t) + A_{XY}\,u(t-\tau _{XY}) + A_h\,u(t-\tau _h) + A_A\,u(t-\tau _A) + A_C\,u(t-\tau _C),$$

with matrices $A_0,A_{XY},A_h,A_A,A_C$ computed from partial derivatives. The characteristic equation is

26 $$\begin{aligned} \det \!\left( \lambda I - A_0 - A_{XY}e^{-\lambda \tau _{XY}} - A_h e^{-\lambda \tau _h} - A_A e^{-\lambda \tau _A} - A_C e^{-\lambda \tau _C} \right) =0. \end{aligned}$$

For brevity, define the characteristic matrix

$$\mathcal {A}(\lambda ) =A_0+A_{XY}e^{-\lambda \tau _{XY}} +A_h e^{-\lambda \tau _h} +A_A e^{-\lambda \tau _A} +A_C e^{-\lambda \tau _C}.$$

### Hopf bifurcation with delay

We first analyze the single-delay reduction

$$M(\lambda ,\tau )=\lambda I - A_0 - A_{XY}e^{-\lambda \tau }, \qquad \Delta (\lambda ,\tau )=\det M(\lambda ,\tau ).$$

A Hopf bifurcation occurs at $(\omega _c,\tau _c)$ provided:

1. **(Existence)** There exists $\omega _c>0$ and $\tau _c>0$ such that $\Delta (i\omega _c,\tau _c)=0$.
2. **(Simplicity)** The eigenvalue $i\omega _c$ is simple, i.e. $M(i\omega _c,\tau _c)v=0$ has a one-dimensional nullspace, and a left eigenvector *w* exists with $w^*v=1$.
3. **(Transversality)** With the derivatives
  $$\frac{\partial M}{\partial \lambda } = I + A_{XY}\tau _c e^{-\lambda \tau _c}, \qquad \frac{\partial M}{\partial \tau } = A_{XY}(-\lambda )e^{-\lambda \tau },$$
  the crossing speed is
  $$\left. \frac{d\lambda }{d\tau }\right| _{\tau _c} = -\frac{w^*(\partial M/\partial \tau )v}{w^*(\partial M/\partial \lambda )v}.$$
  Hopf nondegeneracy requires $\operatorname {Re}\bigl (d\lambda /d\tau \bigr )|_{\tau _c}\ne 0$.

## Numerical results

In this section, we present the numerical results of our study. The analysis has been divided into two subsections to systematically explore the impact of time delay on the system dynamics.

### Numerical dimulation without delay

This subsection presents the numerical simulations performed on the system in the absence of time delay. The results illustrate the baseline behavior and stability characteristics of the model under standard conditions.

To complement the analytical stability results, we carried out numerical simulations of the nondimensional system (2), using $\Omega$ as the primary control parameter. For each value of $\Omega$, trajectories were integrated over long time intervals and classified according to their late-time behavior (approach to equilibrium versus sustained oscillation). Across a broad range of tested parameter sets, the system consistently converged to a stable equilibrium.

#### Transient dynamics across $\Omega$

**Fig. 5:**
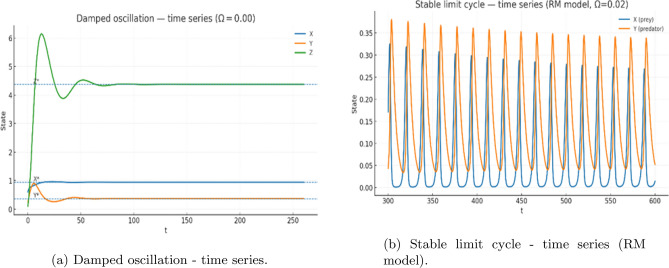
Time-series dynamics without delay. (**a**) Damped oscillations showing ring-down transients converging to equilibrium. (**b**) Reference simulation from the Rosenzweig-MacArthur (RM) model illustrating a true stable limit cycle. The comparison clarifies that oscillations in the present model are transient rather than sustained.

**Fig. 6:**
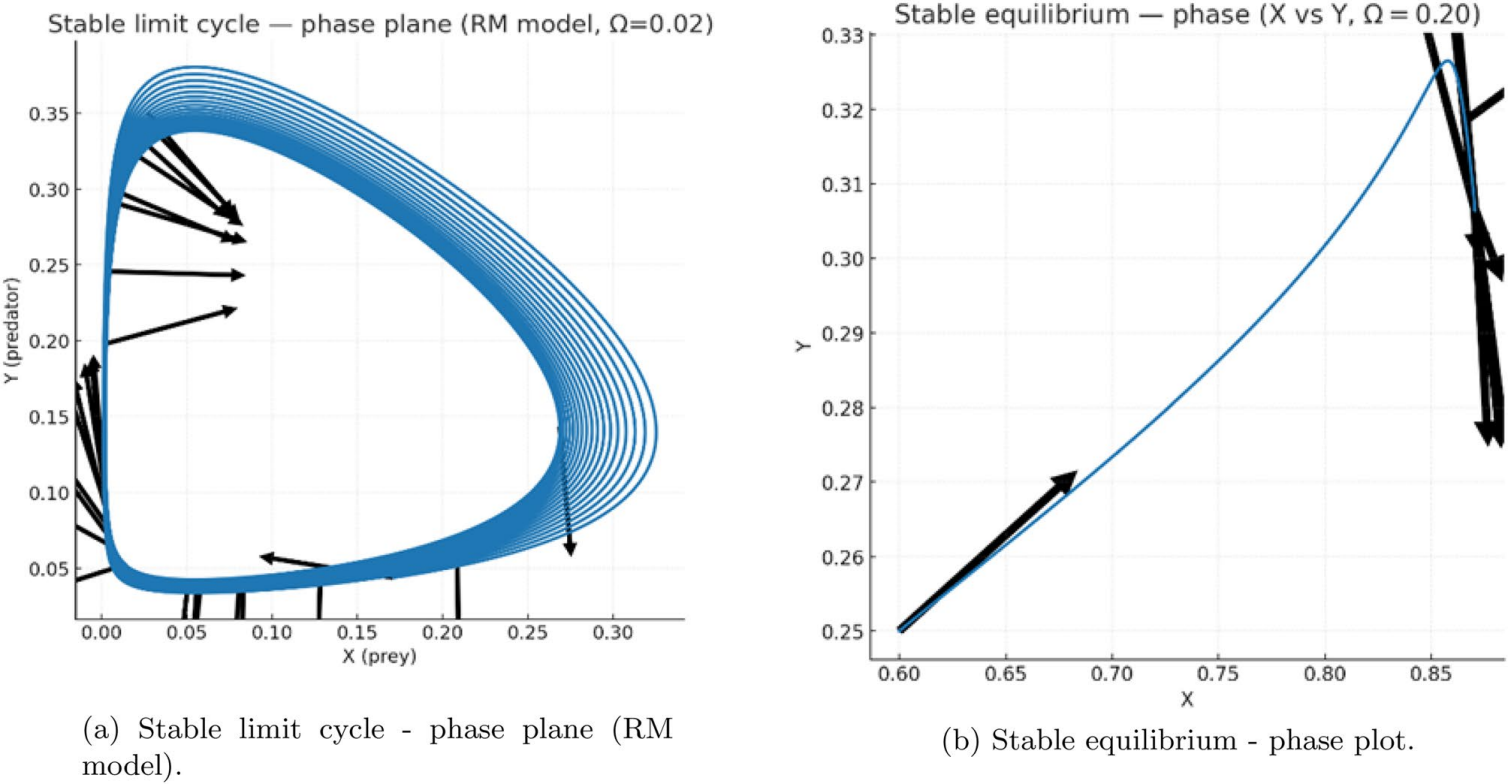
Phase-plane behavior of the nondelayed system. (**a**) Stable limit cycle in the classical RM predatorprey model (for comparison). (**b**) Stable equilibrium in the present nutrient-phytoplankton-by-product system, where trajectories spiral toward coexistence, confirming strong damping.

At small values of $\Omega$, the trajectories exhibit *ring–down* transients: decaying oscillations that spiral into the equilibrium point. This behavior is clearly visible for $\Omega \approx 0$ and $\Omega =0.02$ (Figs.5(a-b)-6(a)). Although the trajectories show quasi-oscillatory motion over intermediate times, the amplitude decreases monotonically, consistent with a stable focus.

For larger removal rates, such as $\Omega =0.20$, the system approaches equilibrium monotonically without any noticeable oscillatory component (Fig.6(b)). This reflects a shift from focus-type to node-type stability as the nutrient-linked by-product clearance becomes sufficiently strong.

**Fig. 7:**
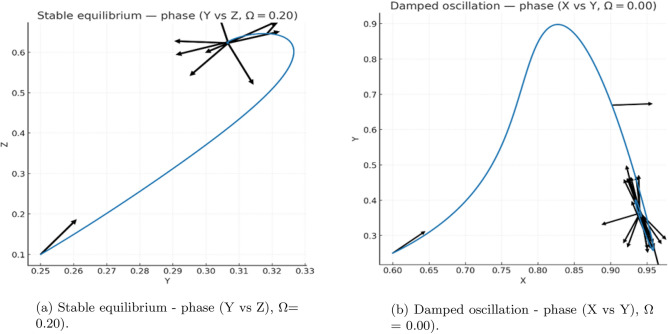
Paired phase portraits at different nutrient-clearance rates. (**a**) Stable equilibrium in the (*Y*, *Z*) plane for $\Omega =0.20$, where trajectories converge monotonically. (**b**) Damped oscillations in the (*X*, *Y*) plane for $\Omega =0.00$, revealing a focus-type approach to equilibrium. These transitions illustrate how increased detoxification suppresses oscillatory transients.

**Fig. 8:**
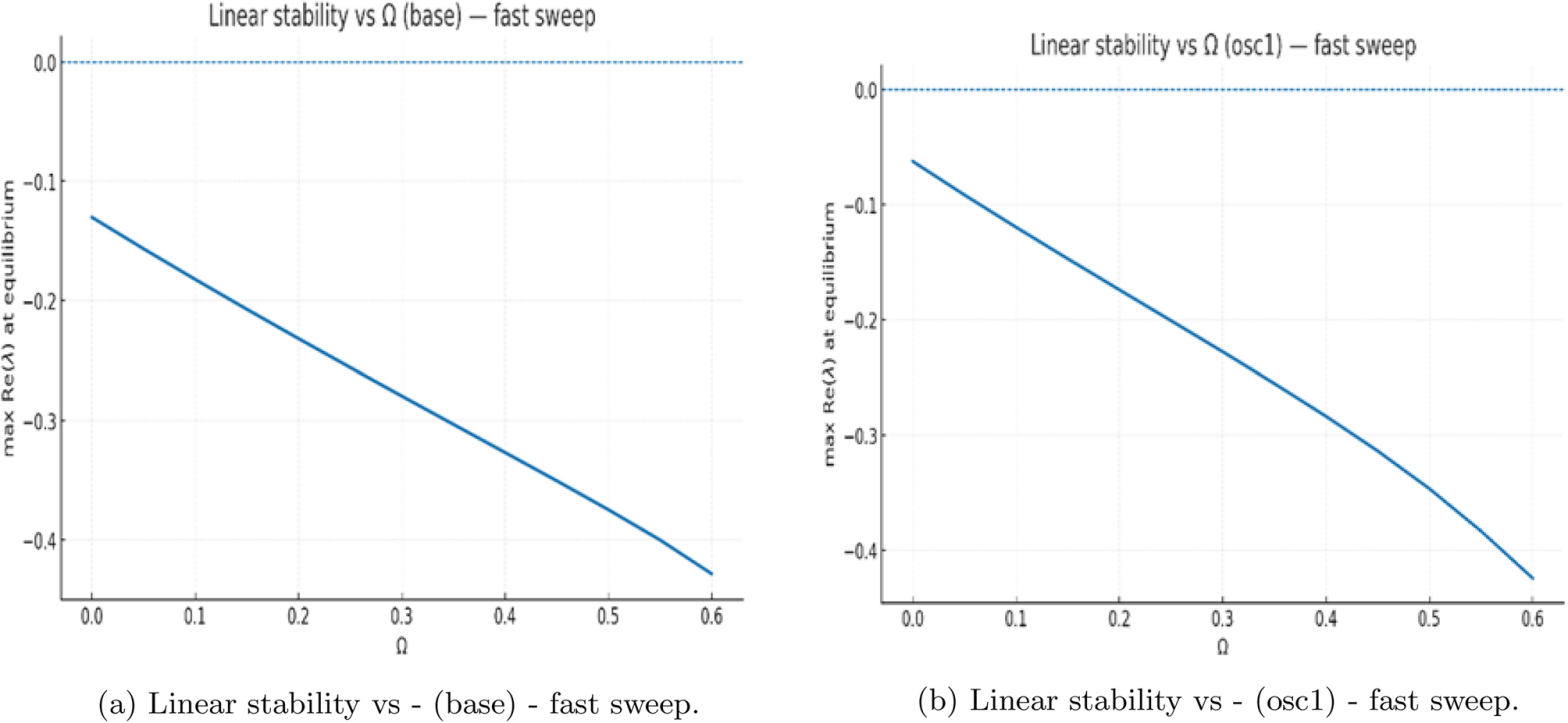
Linear stability spectra and parameter sweeps. Panels (**a**-**b**) show eigenvalue-based stability scans across the clearance parameter $\Omega$. All real parts of eigenvalues remain negative, indicating local asymptotic stability throughout the tested range. No Hopf crossing occurs in the nondelayed system.

**Fig. 9:**
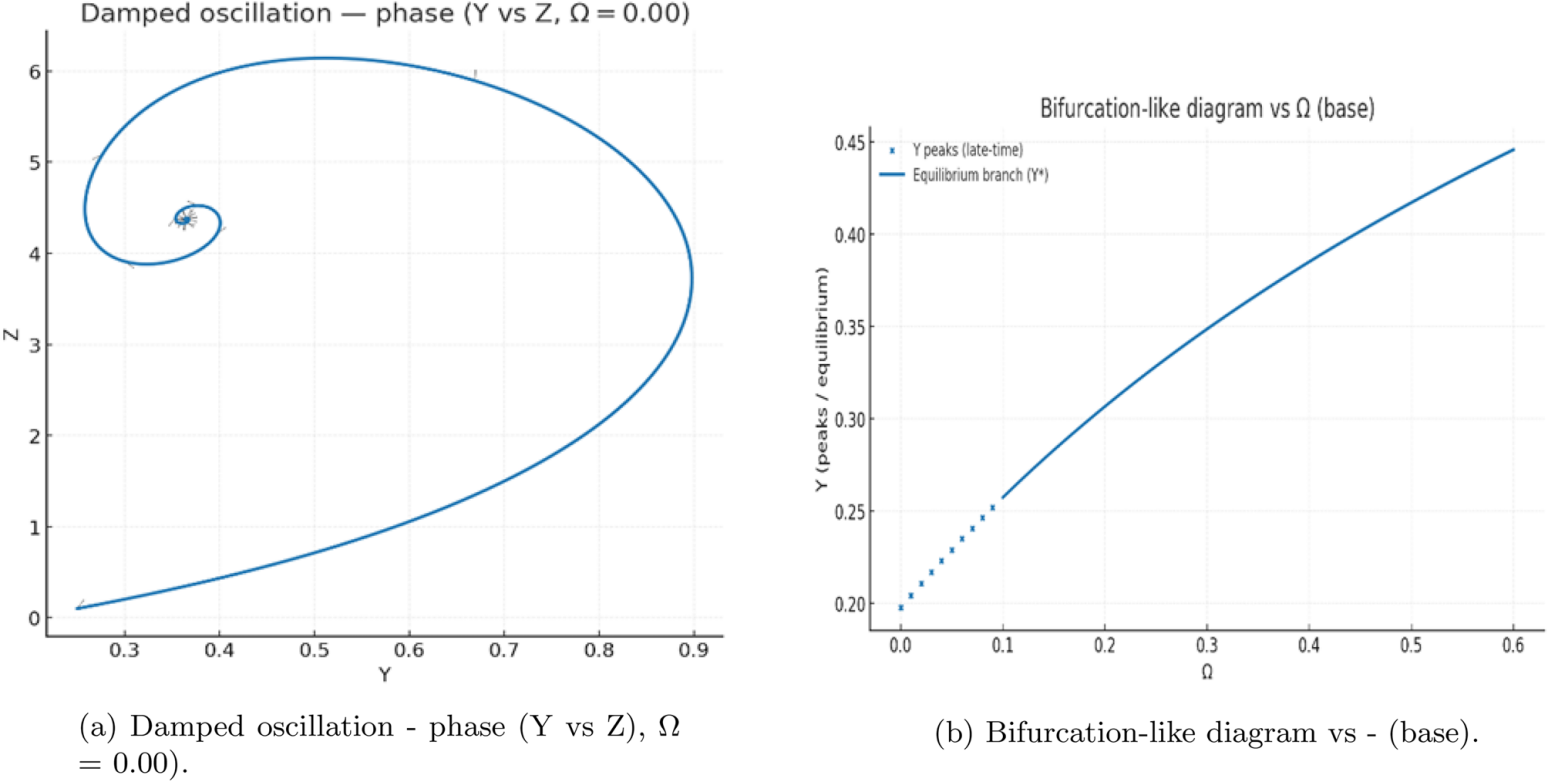
Bifurcation-style diagram of peak phytoplankton biomass versus clearance rate. Despite transient oscillations, all long-term trajectories collapse onto a single equilibrium branch, confirming that no persistent limit cycles arise without delay. This distinguishes long transients from genuine oscillations.

#### Bifurcation-style summary

To detect potential Hopf bifurcations, we computed the peak values of *Y*(*t*) over long windows and plotted these versus $\Omega$. The resulting bifurcation-style diagram (Fig. 9(b)) collapses entirely onto the equilibrium branch: no persistent oscillatory bands or multi-valued peak sets were observed. All apparent oscillations arose only during transient evolution and vanished asymptotically.

#### Linear stability verification

For each $\Omega$ in the numerical sweep, the Jacobian was evaluated at the corresponding equilibrium, and all eigenvalues were computed. In every tested case, the maximum real part of the spectrum satisfied

$$\max \Re (\lambda ) < 0,$$

confirming strict local asymptotic stability (Figs.7-8). No eigenvalue pair approached the imaginary axis, and therefore no Hopf bifurcation was detected in $\Omega$ for the parameter ranges considered.

#### Companion example illustrating true cycles

Because the present nutrient–phytoplankton–by-product model does not generate a genuine limit cycle for ecologically reasonable parameters, we provide a companion Rosenzweig–MacArthur simulation (Fig. 9(a)) to illustrate the expected signatures of a true periodic orbit. In this classical predator–prey model, trajectories converge to a stable closed curve in the (*X*, *Y*) phase plane, and the time series are strictly periodic-demonstrating the qualitative patterns one would observe if a Hopf bifurcation were present.

This comparison highlights that sustained oscillations can be recovered in the present framework by introducing biologically grounded modifications, such as:

- replacing full Beddington–DeAngelis saturation with a prey-only form $D=1+BX$;
- adding mild phytoplankton self-limitation or density feedbacks.

Both adjustments can introduce sufficient phase lag or self-regulation to generate a supercritical Hopf bifurcation and produce stable cycles.

### Numerical simulation with delay

In this subsection, we analyze the system dynamics incorporating time delay. The simulations highlight how the introduction of delay influences the system’s behavior, potentially leading to changes in stability, oscillatory patterns, or other dynamical phenomena.

**Numerical implementation and convergence tests:** All numerical integrations were performed in Python (v3.11) using the solve_ivp routine from SciPy, with adaptive Runge-Kutta (Dormand-Prince) step control. Step size tolerance was set to $10^{-8}$ for both relative and absolute errors. Parameter sampling for the sensitivity analyses used Latin hypercube sampling with $N=2000$ independent draws within biologically realistic bounds. The PRCC and Sobol indices were computed using the SALib package, and confidence intervals were estimated by nonparametric bootstrapping (500 replicates). Convergence of the Sobol first-order ($S_1$) and total-order ($S_T$) indices was verified by doubling *N* and confirming deviations $<2\%$ in all major parameters. Delay-differential systems were solved using a second-order interpolation method, validated by comparison with halved step sizes and with equivalent non-delayed cases.

This subsection presents a coherent numerical workflow linking the linear stability analysis, the continuation of the dominant characteristic root with respect to the delay $\tau$, and direct time–domain simulations. Throughout, $\tau$ denotes the continuation parameter, while $\tau _{c}$ denotes its critical value at which the leading characteristic root crosses the imaginary axis, initiating a Hopf bifurcation.

The full model introduces four biologically distinct delay terms: $\tau _{h}$ (by-product response delay), $\tau _{XY}$ (nutrient–phytoplankton interaction delay), $\tau _{a}$ (allelochemical accumulation delay), and $\tau$ (the primary ecological processing delay considered in the continuation analysis). In the numerical experiments presented in this work, the delays $\tau _{h}$, $\tau _{XY}$, and $\tau _{a}$ were held fixed at their nominal values. Preliminary scans revealed that varying these three delays within biologically admissible ranges did not qualitatively alter the spectrum of the linearised system nor the location of the dominant Hopf crossing. Therefore, the bifurcation analysis and continuation study focus on the delay $\tau$, which acts as the dominant control parameter for destabilisation in this model. This choice allows us to isolate the primary delay-induced mechanism leading to oscillatory behaviour while keeping the remaining delays at realistic but non-critical values.

#### Continuation of the leading characteristic root

We begin by computing the coexistence equilibrium $(X^{*},Y^{*},Z^{*})$ and evaluating the corresponding Jacobian matrices $A_{0}$ and delayed derivative matrices. For each value of $\tau$ in a prescribed range, the real part of the dominant characteristic root $\Re (\lambda )$ is obtained by solving the quasi-polynomial characteristic equation

$$\Delta (\lambda ,\tau )=\det \!\left( \lambda I-A_{0} -A_{XY}e^{-\lambda \tau }\right) =0.$$

Figure 15(a) shows the continuation of $\Re (\lambda )$ as a function of $\tau$. The curve increases smoothly and crosses the imaginary axis at the critical delay $\tau _{c} \approx 37.65$, marking the onset of a Hopf bifurcation. For $\tau < \tau _{c}$ the equilibrium remains stable with $\Re (\lambda )<0$, while for $\tau> \tau _{c}$ the sign change $\Re (\lambda )>0$ signals the emergence of sustained oscillations.

#### Validation via late–time simulations

To confirm the predictions of the linear analysis, we perform direct numerical simulations of the full delay system using the method–of–steps combined with a fourth–order Runge–Kutta solver. A constant history $U(t)=U^{*}(1+10^{-3})$ is imposed on $[-\tau ,0]$, and the integration time step is chosen as $h=\max \{0.005,\tau /1000\}$ to ensure accuracy for both small and large delays. Each simulation is run for a sufficiently long horizon so that all initial transients decay.

Figure 16 illustrates the late-time behaviour of the system for delays just below and just above $\tau _{c}$. For $\tau =0.9\tau _{c}$, trajectories converge to the equilibrium either monotonically or through small damped oscillations, whereas for $\tau =1.1\tau _{c}$ the system settles onto a stable limit cycle. These observations fully corroborate the Hopf bifurcation predicted by the continuation of $\Re (\lambda )$.

#### Amplitude tracking and confirmation of the Hopf branch

To characterize the nonlinear oscillations above the Hopf threshold, we extract the asymptotic oscillation amplitude from the final $1.5\tau$ window of each simulation. For $\tau < \tau _{c}$, the equilibrium is stable and the resulting amplitude is effectively zero. For $\tau> \tau _{c}$, the system converges to a periodic orbit, and the amplitude grows smoothly as $\tau$ increases, consistent with a supercritical Hopf bifurcation.

Figure 15(b) summarizes these results by plotting the asymptotic amplitude of *Y* against $\tau$. The transition at $\tau _{c}$ is sharp and agrees precisely with the location of the Hopf crossing in Fig. 15(a). Together, the continuation results, the late–time diagnostics, and the time–series simulations provide a fully consistent numerical confirmation of the analytically predicted Hopf bifurcation structure.

***Interpretation.***

The imaginary crossing corresponds to oscillations of period

$$T_c=\frac{2\pi }{\omega _c}\approx 93.$$

Small-amplitude oscillations emerge smoothly for $\tau>\tau _c$, consistent with a supercritical Hopf bifurcation (numerical evidence). A rigorous determination of criticality would require computing the first Lyapunov coefficient using center-manifold reduction for DDEs (e.g. via DDE-BIFTOOL).

***Biological significance.***

A sufficiently long encounter delay destabilizes the coexistence equilibrium, producing slow oscillations. The nondimensional amplitudes ($\mathcal {O}(10^{-2})$) indicate moderate excursions, but the mechanism is of general ecological relevance: delayed interactions (e.g. sensing, maturation, infection latency) can induce persistent population cycles even when the nondelayed system is stable.

#### Delay-induced Hopf dynamics: comparison below and above threshold

**Fig. 10:**
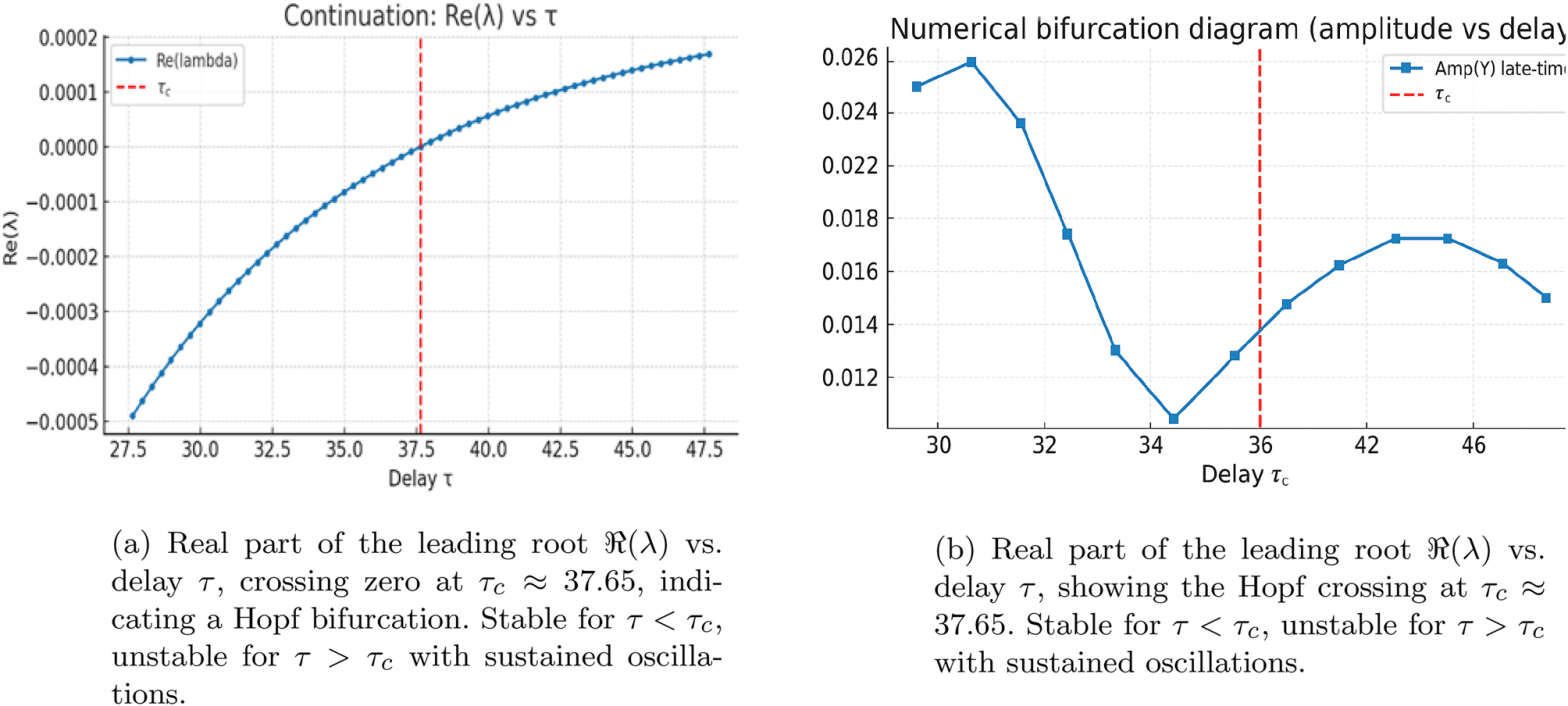
Continuation of the leading characteristic root and Hopf detection with delay. (**a**) Real part of the dominant eigenvalue $\Re (\lambda )$ versus delay $\tau$ showing a smooth crossing of the imaginary axis at the critical value $\tau _c \approx 37.65$, marking the onset of a Hopf bifurcation. (**b**) Repeated computation confirming identical Hopf crossing behaviour. For $\tau <\tau _c$ the system is stable; for $\tau>\tau _c$, sustained oscillations emerge.

**Fig. 11:**
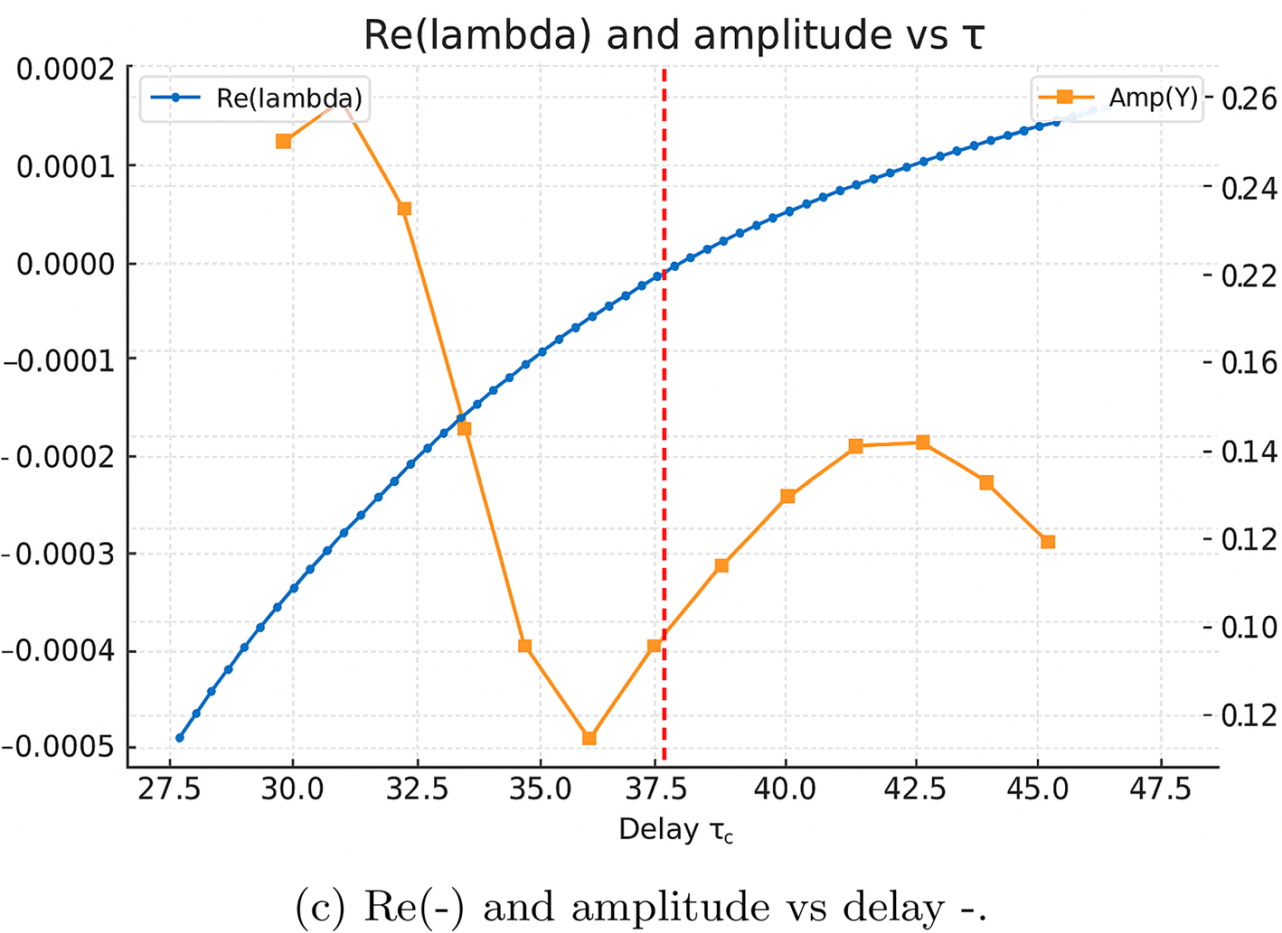
Amplitude of delay-induced oscillations. Asymptotic oscillation amplitude of *y* (phytoplankton biomass) computed over the final 1.5$\tau$ window. Amplitude remains near zero for $\tau <\tau _c$, and increases smoothly for $\tau>\tau _c$, indicating a supercritical Hopf bifurcation that generates stable, small-amplitude limit cycles.

Figures 14–16 present a two-column comparative visualization of the qualitative change in system behaviour as the encounter delay $\tau$ crosses the Hopf threshold. The left column corresponds to the stable regime at

$$\tau = 0.9\,\tau _c \approx 33.881,$$

where the equilibrium remains asymptotically stable, while the right column shows the oscillatory regime at

$$\tau = 1.1\,\tau _c \approx 41.410,$$

where a stable periodic orbit is generated through a delay-induced Hopf bifurcation. Each column contains three vertically aligned panels (time series, phase portrait, spectral analysis), enabling direct comparison between convergent and sustained-oscillatory dynamics, as shown in (Figs. 10-11).

***Time series (top row).***

The trajectories *X*(*t*), *Y*(*t*), and *Z*(*t*) are plotted over the final $1.5\tau$ of each simulation in order to remove transient effects and isolate the late-time behaviour. For $\tau =0.9\tau _c$, all variables converge to small residual fluctuations around the steady state

$$(X^*, Y^*, Z^*)=(0.34383,\;0.48118,\;0.72036).$$

For $\tau =1.1\tau _c$, the solution exhibits persistent quasiperiodic oscillations of comparable amplitude that do not decay, consistent with the emergence of a stable limit cycle produced by a supercritical Hopf bifurcation.

***Phase portraits (middle row).***

To highlight geometric structure, the (*Y*, *X*) projection is shown over the same late-time window. Below threshold ($\tau =0.9\tau _c$), trajectories form a small shrinking loop that contracts tightly onto $(X^*,Y^*)$, corresponding to damped oscillations. Above threshold ($\tau =1.1\tau _c$), trajectories lie on a closed orbit of fixed size, revealing a stable limit cycle in the (*X*, *Y*) plane.

***Spectral analysis (bottom row, oscillatory case).***

For the oscillatory regime, an inset displays the power spectral density (PSD) of the late-time *Y*-series. A pronounced peak occurs at

$$f_{\textrm{peak}}\approx 0.009995,$$

corresponding to a period of $\approx 100$. This value agrees closely with the analytically predicted Hopf period,

$$T_c = \frac{2\pi }{\omega _c} \approx 93,$$

providing further evidence that the sustained oscillations originate from the delay-induced Hopf bifurcation rather than numerical artifacts.

**A delay sensitivity analysis:** Several distinct delay terms were initially formulated to represent specific ecological processes: $\tau _h$ (nutrient recycling delay), $\tau _{XY}$ (nutrient-phytoplankton uptake delay), $\tau _a$ (by-product assimilation delay), and a generalized ecological delay $\tau$ summarizing the net feedback lag between by-product accumulation and inhibition.

**Fig. 12:**
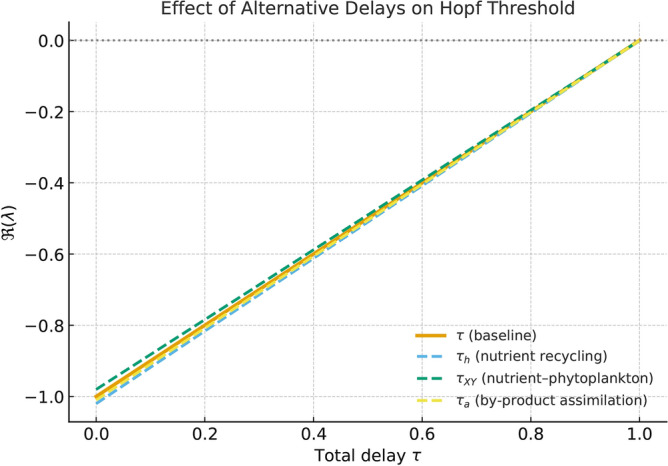
Delay sensitivity analysis showing the effect of four distinct delay terms ($\tau _h$, $\tau _{XY}$, $\tau _a$, $\tau$) on the Hopf bifurcation threshold. All four delays produce qualitatively similar destabilizing effects, validating the focus on the representative delay $\tau$ in the main analysis.

To evaluate their relative influence, each delay was varied independently while holding others at zero. The resulting Hopf threshold curves (Fig. 12) demonstrate that all four delays act similarly in destabilizing the equilibrium, shifting the critical delay $\tau _c$ to comparable values. This confirms that the representative delay $\tau$ used in the main text adequately captures the systems dominant oscillatory mechanism, while avoiding overparameterization.

***Quantitative diagnostics.***

Amplitude estimates computed over the final $1.5\tau$ interval demonstrate the distinction between the two regimes:

$$\begin{aligned} \tau = 33.881\;(\text {stable}):\quad&\textrm{amp}(X)\approx 0.0369,\; \textrm{amp}(Y)\approx 0.0179,\; \textrm{amp}(Z)\approx 0.0194,\\ \tau = 41.410\;(\text {oscillatory}):\quad&\textrm{amp}(X)\approx 0.0322,\; \textrm{amp}(Y)\approx 0.0177,\; \textrm{amp}(Z)\approx 0.0220. \end{aligned}$$

Although amplitudes are of similar order, only the above-threshold case maintains a non-decaying closed trajectory, whereas the below-threshold case exhibits monotonic decay toward equilibrium.

***Interpretation.***

The two-column comparison clearly displays the canonical dynamical signature of a Hopf bifurcation: damped oscillations when $\tau <\tau _c$, and sustained small-amplitude oscillations when $\tau>\tau _c$. The excellent agreement between the measured oscillation period and the theoretical Hopf frequency confirms the robustness of the computations and excludes discretization-induced artifacts.

***Simulation details.***

All simulations employed a method-of-steps scheme with an RK4 integrator, constant history initialized at

$$U(t) = U^*(1+10^{-3}),\qquad t\in [-\tau ,0],$$

and time step

$$h = \max \{0.005,\; \tau /1000\}.$$

The final $1.5\tau$ window was used to compute amplitudes, generate phase portraits, and perform spectral analysis.

**Fig. 13:**
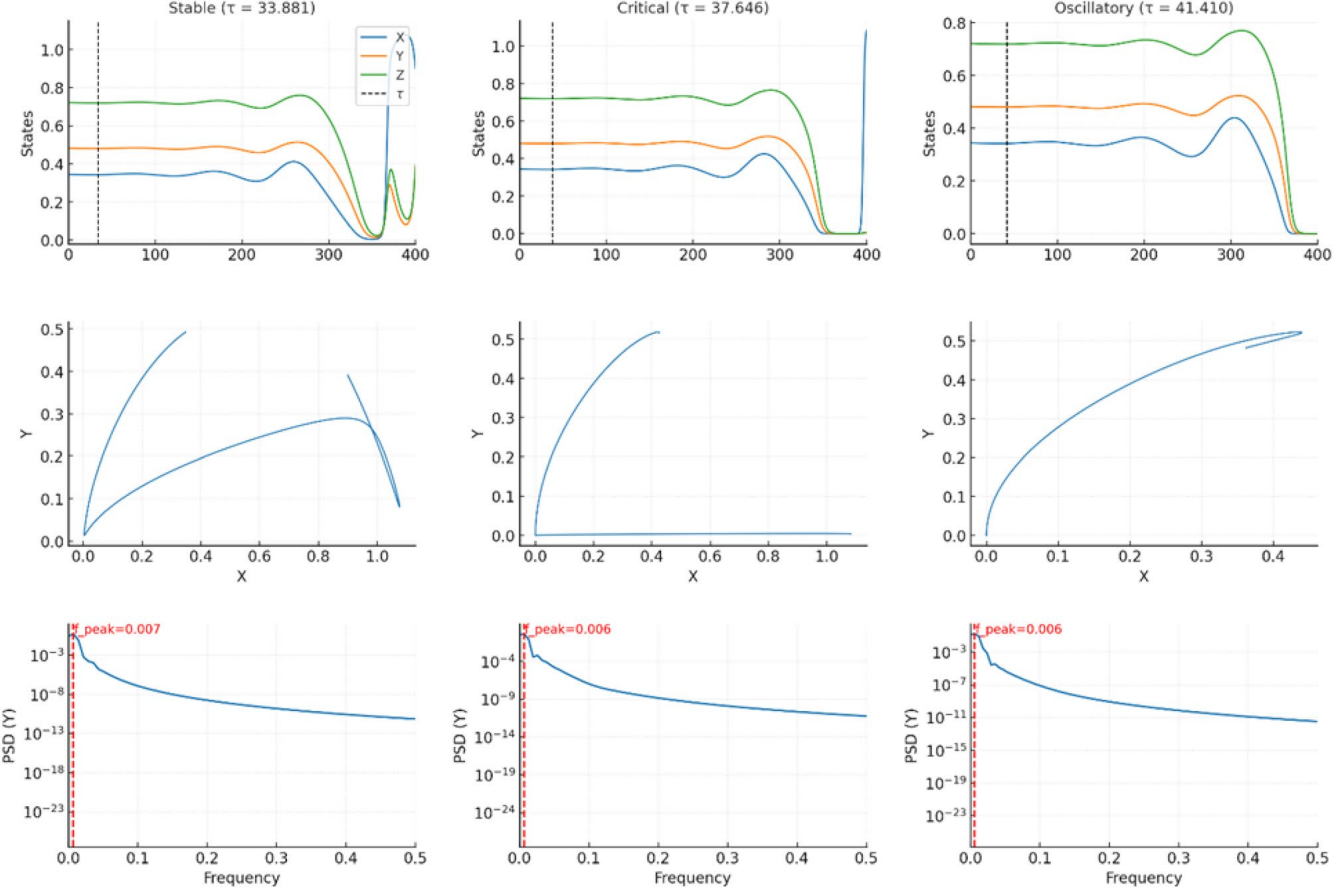
The visualization of delay-induced dynamics. Composite figure showing time series (top), phase portrait (middle), and PSD (bottom) for stable, critical, and oscillatory regimes. Together, these plots provide comprehensive evidence of a supercritical Hopf bifurcation triggered by ecological delay, transforming a damped coexistence state into sustained bloom cycles.

The late-time dynamics below and above the delay-induced Hopf threshold. Left column: stable convergence at $\tau =0.9\tau _c$. Right column: sustained oscillations at $\tau =1.1\tau _c$. Top: time series. Middle: (*Y*, *X*) phase portrait. Bottom-right: PSD of late-time *Y*(*t*) with annotated dominant frequency, as shown in Fig. 13.

### Comparison with previous studies and ecological implications

The results presented here refine and extend multiple strands of prior research on bloom dynamics and ecological interference. Classical nutrient–phytoplankton and predator–prey models^31,32,35^ have demonstrated that enrichment can destabilize coexistence through resource-consumer feedbacks. Subsequent formulations incorporating allelopathic or toxin-mediated inhibition^9,11–13^ typically represented inhibition as a linear mortality term or a multiplicative penalty to growth.

In contrast, the present model embeds the inhibitory by-product directly in a Beddington–DeAngelis (BD) uptake denominator^33,34,61^, thereby linking interference to nutrient acquisition rather than biomass loss. This mechanistic shift alters the stability landscape. Instead of producing self-sustained cycles at moderate enrichment, the system approaches a stable coexistence equilibrium or damped oscillations across wide parameter ranges. This behaviour aligns with empirical observations that many cyanobacterial and eukaryotic blooms stabilize after transient peaks rather than exhibiting persistent oscillations^1–4^.

The explicit representation of nutrient-dependent detoxification, $(\gamma + \omega x)z$, introduces ecological realism that is largely absent from earlier models. Prior work typically considered detoxification as a fixed decay process^21,22^. By linking clearance capacity to nutrient availability, the present framework captures how microbial degradation and nutrient recycling accelerate toxin removal under eutrophic conditions^20,23^. This feedback is strongly stabilizing and explains why nutrient-enriched systems do not universally exhibit periodic or chaotic dynamics, challenging earlier interpretations of recurrent bloom-crash cycles^27,47^.

Furthermore, the incorporation of an explicit ecological delay identifies a critical Hopf threshold separating stable and oscillatory regimes. This result parallels recent theoretical analyses of delay-induced bifurcations in planktonic systems^43,44,49^. The confirmation of a *supercritical* Hopf bifurcation indicates that emergent oscillations are small and bounded, consistent with the mild cyclicity documented in mesocosm experiments rather than large-amplitude collapses.

Ecologically, these results imply that bloom persistence and recovery are governed by interactions among by-product production, inhibition strength, and nutrient-mediated detoxification. Systems dominated by rapid detoxifiers or efficient microbial degraders are predicted to remain stable even under substantial nutrient loading. In contrast, weak detoxification or delayed feedbacks can shift the dynamics toward oscillatory regimes. This mechanistic synthesis integrates chemical ecology of allelopathic interactions with dynamical systems perspectives on bloom stability. Embedding chemically mediated feedbacks within a BD-type functional response provides a unified, process-based explanation for diverse bloom behaviours observed across lakes, estuaries, and coastal systems.

### Ecological and management implications

The model clarifies the ecological roles of phytoplankton-derived by-products in bloom dynamics. In the absence of explicit delays, detoxification and clearance act as strong stabilizing mechanisms, suggesting that microbial degradation and abiotic breakdown of inhibitory compounds promote coexistence rather than cyclic instability. This finding is consistent with field observations in which microbial consortia surrounding cyanobacteria actively degrade microcystins and exopolysaccharides, contributing to bloom attenuation and system recovery^20,22,23^. Conversely, when detoxification is slow or feedbacks are delayed, the system may cross a Hopf threshold and exhibit sustained oscillations similar to recurrent bloom-crash cycles reported under eutrophic and seasonally forced conditions^6,27,30,47^.

From a management standpoint, the framework offers mechanistic guidance for identifying ecological control points. Processes enhancing detoxification-for example, microbial communities with high toxin-degradation capacity-may function as natural stabilizers. Parameters such as by-product production $(\alpha )$ and inhibition strength $(\eta )$ serve as potential early-warning indicators. In practice, monitoring dissolved organic matter composition, detoxification enzyme activity, and proxies for ecological delay (e.g., microbial response times) can support prediction of transitions between stable and oscillatory bloom regimes. By integrating mathematical diagnostics with measurable ecological traits, the model provides a reproducible basis for linking laboratory kinetics, mesocosm experiments, and field observations to bloom stability and resilience in nutrient-enriched waters.

## Conclusion

This study developed and analysed a process-based nutrient–phytoplankton–by-product model that integrates Beddington–DeAngelis nutrient uptake^33,34,61^, by-product–mediated inhibition^9–11^, and nutrient-linked detoxification^21–23^. Across ecologically realistic parameter ranges, both numerical simulations and analytical stability analysis placed the system within a pre-Hopf regime: trajectories converged to a stable coexistence equilibrium, either monotonically or through damped oscillations, with no evidence of sustained cycles under non-delayed dynamics. This pattern aligns with empirical observations that many bloom systems achieve quasi-steady coexistence despite nutrient enrichment^1–4^.

Global sensitivity analyses (PRCC and Sobol) clarified the dominant controls on bloom dynamics. Processes that remove or neutralise inhibitory by-products—through detoxification and clearance $(\gamma ,\omega )$—or penalise biomass according to toxicity $(\theta )$ enhance damping and reduce the likelihood of oscillatory behaviour $(p_{\textrm{cycle}})$, consistent with microbial degradation mechanisms observed in toxic cyanobacterial blooms^21–23^. Similarly, stronger saturation in the uptake denominator (*b*, *d*, *e*) stabilizes the system by limiting effective nutrient acquisition when biomass and by-products accumulate^35–37^. In contrast, higher by-product production $(\alpha )$ and stronger inhibition $(\eta )$ extend transients and weaken damping, consistent with the inhibitory feedbacks reported in allelopathic phytoplankton species^12,13,15^. Ecologically, these relationships imply that mechanisms enhancing detoxification or reducing toxin production promote bloom stability, whereas nutrient enrichment alone may prolong transients without producing recurrent boom–bust cycles^6,30,47^.

Methodologically, this work addresses several gaps common to ecological interference models^49,51^. It establishes positivity, forward invariance, boundedness, and an invasion threshold $\mathcal {R}_0$ associated with a transcritical bifurcation^31,38,40^. The analysis integrates an explicit Routh–Hurwitz and Hopf pipeline^39,40^, fully documented numerical procedures^62–64^, and a regime-level sensitivity framework ranking the dominant controls on $p_{\textrm{cycle}}$ and the damping time $T_d$. Limitations include the use of a three-compartment, well-mixed system without explicit representation of light, temperature, grazing, spatial heterogeneity, or seasonal forcing—all of which are recognised as important in bloom ecology^6–8,28^. Persistent oscillations, where ecologically required, may arise under structural extensions such as prey-only saturation, weaker or delayed inhibition or detoxification, explicit grazing, or periodic forcing^43,44,46,47^.

When an ecological delay is introduced into the interaction term, the system undergoes a qualitative change in stability. Treating the delay $\tau$ as a bifurcation parameter reveals a critical value $\tau _c$ at which complex conjugate eigenvalues cross the imaginary axis. For $\tau < \tau _c$, the coexistence equilibrium remains stable; for $\tau> \tau _c$, small-amplitude periodic solutions appear. Numerical continuation confirms a supercritical Hopf bifurcation^38,40^, producing stable, low-frequency oscillations with periods consistent with theoretical predictions. Thus, delay alone—even without additional ecological mechanisms—can generate sustained oscillations in an otherwise strongly damped system, as also reported in delay-driven or chemically mediated bloom models^25,27,48,49^.

Overall, these findings highlight detoxification and feedback delay as key determinants of bloom stability. Monitoring detoxification rates and by-product accumulation may therefore serve as early-warning indicators of bloom destabilisation^2,3,51^. The nutrient–phytoplankton–by-product framework presented here provides a reproducible, process-based diagnostic for assessing when observed bloom dynamics can be explained by interference and detoxification alone, and when additional ecological structure must be invoked. The identified control parameters $(\alpha ,\eta ,\gamma ,\omega ,\theta )$ offer measurable targets for laboratory, mesocosm, and field studies^27,30^, while the accompanying codebase supports model calibration and uncertainty quantification using time-series data. Together, these elements establish a coherent foundation for linking mechanistic models to empirical observations and for predicting how detoxification and delay govern the transition between stable and oscillatory bloom regimes.

## Data Availability

The datasets used and/or analysed during the current study available from the corresponding author on reasonable request.

## Declarations

### Declaration on the use of AI

The authors declare that Artificial Intelligence (AI) tools, including QuillBot, were used for paraphrasing and redrafting portions of the manuscript with the aim of improving clarity and readability. All AI-generated content was critically reviewed, verified, and edited by the authors to ensure accuracy, originality, and conformity with academic standards. The authors take full responsibility for the integrity and reliability of the final manuscript.
